# *N*-Methyl-d-Aspartate (NMDA) Receptor Blockade Prevents Neuronal Death Induced by Zika Virus Infection

**DOI:** 10.1128/mBio.00350-17

**Published:** 2017-04-25

**Authors:** Vivian V. Costa, Juliana L. Del Sarto, Rebeca F. Rocha, Flavia R. Silva, Juliana G. Doria, Isabella G. Olmo, Rafael E. Marques, Celso M. Queiroz-Junior, Giselle Foureaux, Julia Maria S. Araújo, Allysson Cramer, Ana Luíza C. V. Real, Lucas S. Ribeiro, Silvia I. Sardi, Anderson J. Ferreira, Fabiana S. Machado, Antônio C. de Oliveira, Antônio L. Teixeira, Helder I. Nakaya, Danielle G. Souza, Fabiola M. Ribeiro, Mauro M. Teixeira

**Affiliations:** a Immunopharmacology Lab, Department of Biochemistry and Immunology, Institute of Biological Sciences (ICB), Universidade Federal de Minas Gerais (UFMG), Minas Gerais, Brazil; b Host-Interaction Microorganism Lab, Department of Microbiology, Institute of Biological Sciences (ICB), Universidade Federal de Minas Gerais (UFMG), Belo Horizonte, Minas Gerais, Brazil; c Neurobiochemistry Lab, Department of Biochemistry and Immunology, Institute of Biological Sciences (ICB), Universidade Federal de Minas Gerais (UFMG), Minas Gerais, Brazil; d Cardiac Biology Lab, Department of Morphology, Institute of Biological Sciences (ICB), Universidade Federal de Minas Gerais (UFMG), Belo Horizonte, Minas Gerais, Brazil; e Immunoregulation of Infectious Disease Lab, Department of Biochemistry and Immunology, Institute of Biological Sciences (ICB), Universidade Federal de Minas Gerais (UFMG), Minas Gerais, Brazil; f Virology Lab, Department of Virology, Universidade Federal da Bahia (UFBA), Salvador, Bahia, Brazil; g Neuropharmacology Lab, Department of Pharmacology, Institute of Biological Sciences (ICB), Universidade Federal de Minas Gerais (UFMG), Belo Horizonte, Minas Gerais, Brazil; h Neuropsychiatry Program, Department of Psychiatry and Behavioral Sciences, McGovern Medical Houston, University of Texas Health Science Center at Houston, Houston, Texas, USA; i Metabolomics Applied to Health Lab, Department of Clinical Analyses and Toxicology, School of Pharmaceutical Science, Universidade de Sao Paulo (USP), Sao Paulo, Brazil; University of California, Irvine

**Keywords:** NMDA receptor, Zika virus, intraocular pressure, memantine, microgliosis, mouse model, neuronal death

## Abstract

Zika virus (ZIKV) infection is a global health emergency that causes significant neurodegeneration. Neurodegenerative processes may be exacerbated by *N*-methyl-d-aspartate receptor (NMDAR)-dependent neuronal excitoxicity. Here, we have exploited the hypothesis that ZIKV-induced neurodegeneration can be rescued by blocking NMDA overstimulation with memantine. Our results show that ZIKV actively replicates in primary neurons and that virus replication is directly associated with massive neuronal cell death. Interestingly, treatment with memantine or other NMDAR blockers, including dizocilpine (MK-801), agmatine sulfate, or ifenprodil, prevents neuronal death without interfering with the ability of ZIKV to replicate in these cells. Moreover, *in vivo* experiments demonstrate that therapeutic memantine treatment prevents the increase of intraocular pressure (IOP) induced by infection and massively reduces neurodegeneration and microgliosis in the brain of infected mice. Our results indicate that the blockade of NMDARs by memantine provides potent neuroprotective effects against ZIKV-induced neuronal damage, suggesting it could be a viable treatment for patients at risk for ZIKV infection-induced neurodegeneration.

## IMPORTANCE

Zika virus (ZIKV) infection is a global health emergency associated with serious neurological complications, including microcephaly and Guillain-Barré syndrome. Infection of experimental animals with ZIKV causes significant neuronal damage and microgliosis. Treatment with drugs that block NMDARs prevented neuronal damage both *in vitro* and *in vivo*. These results suggest that overactivation of NMDARs contributes significantly to the neuronal damage induced by ZIKV infection, and this is amenable to inhibition by drug treatment.

## INTRODUCTION

*Zika virus* (ZIKV) is a mosquito-borne pathogen that poses significant public health concerns due to the recent rapidly expanding outbreak. It is an emerging pathogen that belongs to the *Flavivirus* genus and *Flaviviridae* family, along with other clinically important arboviruses, including *Dengue virus* (DENV), *Chikungunya virus* (CHIKV), and *West Nile virus* (1–3). Since its first isolation in 1947 from a rhesus monkey in the Ziika Forest in Uganda (4), sporadic and benign human cases of ZIKV infection, most of them asymptomatic, have been reported in Africa and Asia (5), until the first serious outbreak that occurred in the population of Yap Island (Micronesia) in 2007 (6). This was followed by a major outbreak in French Polynesia in 2013 (7). Recently, ZIKV was introduced to the Western Hemisphere, causing an ongoing epidemic in South America, with millions of infections across Brazil, Colombia, and Venezuela (6, 8). The first case of ZIKV infection in Brazil was reported in May 2015 (9), and ZIKV infection has been associated with severe neurological complications, including microcephaly and ophthalmological alterations, such as severe macular neuroretinal atrophy and foveal reflex loss in infants born from ZIKV-infected mothers (10–13), uveitis (14), and direct virus-induced inflammatory polyneuropathy and Guillain-Barré syndrome (GBS) in adults (15, 16). As a result, on February 2016, the World Health Organization announced that the ZIKV outbreak was a Public Health Emergency of International Concern (17). However, so far there is no available vaccine and treatment is only supportive (18).

The pathogenesis of ZIKV infection remains poorly understood and involves a complex interplay between viral and host factors. Recent studies have shown that ZIKV has extensive tropism to the central nervous system (CNS) and causes significant neurodegeneration, especially of neural progenitor cells (19–22). These neurodegenerative effects appear to account for the neurological disorders associated with ZIKV infection (7, 12, 23).

Glutamate is the main excitatory neurotransmitter in the brain and plays a pivotal role during neurodegenerative processes (24–26). There are two types of glutamate receptors: ionotropic and metabotropic (27). Several studies indicate that glutamatergic overstimulation via activation of ionotropic glutamate receptors leads to excitotoxicity, which promotes neuronal calcium overload and, consequently, neurodegeneration (28). Here, we hypothesize that *N*-methyl-d-aspartate receptor (NMDAR) blockade by memantine can avoid the death of nearby neurons and decrease neurodegeneration and neuroinflammation associated with ZIKV infection. For instance, the NMDAR antagonist memantine has been approved by international regulatory agencies for the treatment of Alzheimer’s disease (AD) (26). Moreover, the neuroprotective properties of memantine have been described in other contexts, such as cerebral ischemia (29). In AIDS, memantine appears to inhibit neuronal damage promoted by the viral protein gp120 (30–33).

Here, using relevant *in vitro* and *in vivo* settings, we investigated whether NMDAR blockade could prevent neurodegeneration induced by ZIKV infection. Our data show that blocking NMDARs prevents neuronal death induced by ZIKV, suggesting that memantine may be a useful therapy to prevent neurological disorders in ZIKV-infected patients.

## RESULTS

### Characterization of clinical, inflammatory, and virological aspects of a Brazilian ZIKV strain in WT (SV129) and IFN-α/βR^−/−^ mice.

**FIG 1:**
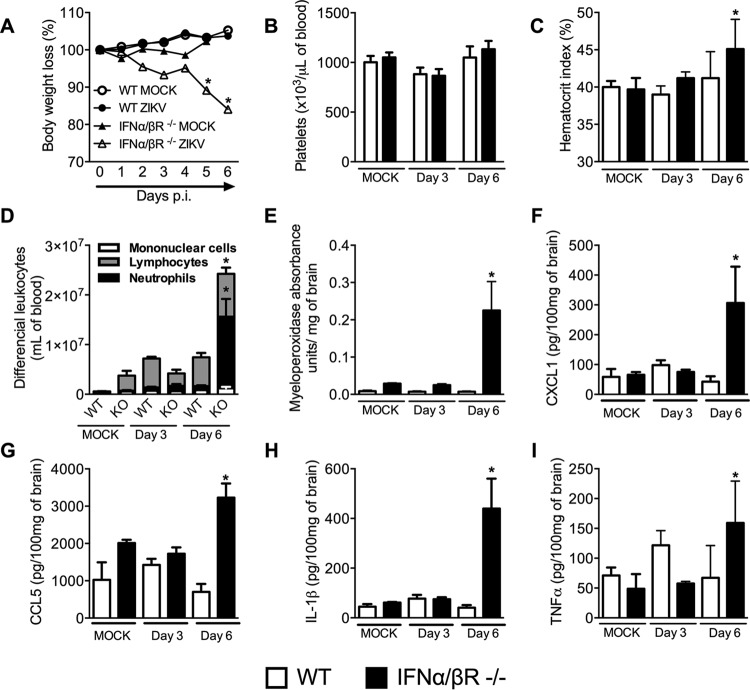
Characterization of the disease parameters in WT and IFN-α/βR^−/−^ mice infected with a Brazilian strain of Zika virus. (A) WT and IFN-α/βR^−/−^ mice (*n =* 9 to 11) were inoculated i.v. with 4 × 10^5^ PFU of a Brazilian ZIKV strain (HS-2015-BA-01), and change in body weight was analyzed daily. Results are expressed as the percentage of initial weight loss. (B to I) Three or 6 days (peak of disease) after ZIKV inoculation, mice were culled and blood and tissues collected for the following analyses: (B) number of platelets, shown as platelets × 10^3^/ml of blood; (C) hematocrit index, expressed as percentage of volume occupied by red blood cells; (D) total and differential cell counts on blood, represented as the number of differential cell counts (leukocytes, mononuclear cells, and neutrophils) normalized by the percentage of total cell counts; (E) neutrophil influx to the brain; (F to I) concentrations of CXCL1, CCL5, IL-1β, and TNF-α in mouse brain, shown as picograms per 100 mg of brain tissue. All results are expressed as the mean ± standard error of the mean (SEM) and are representative of at least two independent experiments. *, *P* < 0.05 compared to control uninfected mice (MOCK). WT, SV129 mice; KO (knockout), IFN-α/βR^−/−^ mice. p.i., postinfection.

Recent studies have reported successful ZIKV infection of mice lacking type I interferon (IFN-α/β) responses, both type I and type II IFN responses, or other components of the innate immune system (34–36). Here, we decided first to characterize the systemic infection induced by a Brazilian isolate of ZIKV, HS-2015-BA-01, in adult wild-type (WT) mice (SV129), type I interferon receptor-deficient (IFN-α/βR^**−/−**^) mice, and type II interferon receptor-deficient (IFN-γR^**−/−**^) mice. In all experiments, uninfected control (mock-infected) mice were inoculated with the supernatant of a cell suspension from the mosquito C6/36 *Aedes albopictus* culture medium, which caused no clinical or biochemical alterations in comparison to uninoculated mice (data not shown). Systemic infection of IFN-α/βR^**−/−**^ mice with 4 × 10^5^ PFU of ZIKV induced lethality that was observed around the 6th and 7th days after ZIKV inoculation (see Fig. S1A in the supplemental material). Since around 80% of the IFN-α/βR^**−/−**^ infected mice succumbed to the infection on day 6, all subsequent experiments were conducted at this time point. No lethality was observed for WT and IFN-γR^**−/−**^ mice after ZIKV inoculation (Fig. S1A). Disease manifestations, characterized by appearance of clinical signs such as ruffled fur, ataxia, partial or complete hind limb weakness or paralysis, and massive body weight loss (Fig. 1A), starting from day 5 after infection, were detectable only in IFN-α/βR^**−/−**^ ZIKV-infected group. In contrast, MOCK-infected WT and IFN-α/βR^**−/−**^ mice or WT infected mice did not present any body weight loss or signs of disease (Fig. 1A). Infection of IFN-γR^**−/−**^ mice, even with higher inocula, did not induce any signs of disease manifestation, as shown by the absence of lethality (Fig. S1A) or body weight loss (Fig. S1B), indicating that type II IFN deficiency alone is not sufficient to induce disease or death after ZIKV infection.

10.1128/mBio.00350-17.1 FIG S1  Type I interferons are essential to control ZIKV infection, and IFN-γ signaling is not important. (A) WT, IFN-α/βR^−/−^, and IFN-γR^−/−^ mice (*n =* 8 mice per group) were inoculated with 4 × 10^5^ PFU of a Brazilian ZIKV strain (HS-2015-BA-01), and lethality rates were analyzed every 12 h for 14 days. Results are shown as percentage of survival. (B) IFN-γR^−/−^ mice (*n =* 6 mice per group) were inoculated with 1 × 10^6^, 1 × 10^7^, or 1 × 10^8^ PFU of a Brazilian ZIKV strain (HS-2015-BA-01). Change in body weight was analyzed daily for 11 days. Results are expressed as percentage of initial weight loss. p.i., postinfection. Download FIG S1, TIF file, 0.3 MB. Copyright © 2017 Costa et al. 2017 Costa et al. This content is distributed under the terms of the Creative Commons Attribution 4.0 International license.

Next, we performed a series of experiments to characterize the clinical and inflammatory aspects at days 3 and 6 (peak of disease manifestation) after ZIKV infection in WT and IFN-α/βR^**−/−**^ mice. No significant changes in platelet levels (Fig. 1B), hematocrit index (Fig. 1C), or total and differential blood counts (Fig. 1D) were detected in ZIKV-infected WT mice compared to mock-infected mice. Levels of inflammatory parameters represented by levels of myeloperoxidase (MPO) activity, indicative of neutrophil recruitment to the brain (Fig. 1E), or production of chemokines or cytokines, as assessed by levels of CXCL1 (Fig. 1F), CCL5 (Fig. 1G), interleukin-1β (IL-1β) (Fig. 1H), and tumor necrosis factor alpha (TNF-α) (Fig. 1I) in the brain of ZIKV-infected WT mice, at both 3 and 6 days after ZIKV inoculation, were similar to those of mock-infected mice (Fig. 1E to I). However, significant alterations of all of the parameters mentioned were detected in IFN-α/βR^**−/−**^ mice at day 6 after ZIKV inoculation (Fig. 1C to I), with exception of platelet levels (Fig. 1B). More specifically, increased hematocrit index (Fig. 1C), elevated total and differential blood leukocyte counts, especially neutrophil numbers (Fig. 1D), and very high levels of MPO in brain tissue (Fig. 1E) were detected in ZIKV-infected IFN-α/βR^**−/−**^ mice. Additionally, elevated levels of the inflammatory mediators CXCL1 (Fig. 1F), CCL5 (Fig. 1G), IL-1β (Fig. 1H), and TNF-α (Fig. 1I) were detected in the brain of ZIKV-infected IFN-α/βR^**−/−**^ mice. Of note, no changes in the levels of the following mediators were recorded: brain-derived neurotrophic factor (BDNF), nerve growth factor (NGF), IL-10, or IL-6 (see Fig. S2 in the supplemental material). Therefore, our results show that infection of IFN-α/βR^**−/−**^ mice with ZIKV induces several clinical and systemic inflammatory manifestations that impact directly on the CNS of these mice.

10.1128/mBio.00350-17.2 FIG S2  Concentration of inflammatory mediators in the brain of WT and IFN-α/βR^−/−^ mice after ZIKV infection. (A to D) IFN-α/βR^−/−^ mice (*n =* 5 to 7 mice per group) were inoculated i.v. with 4 × 10^5^ PFU of a Brazilian ZIKV strain (HS-2015-BA-01). Concentrations of brain-derived neurotrophic factor (BDNF [A]), nerve growth factor (NGF [B]), IL-10 (C), and IL-6 (D) in the brain of mice were quantified by enzyme-linked immunosorbent assay (ELISA). Results are shown as picograms per 100 mg of brain tissue. MOCK, uninfected. All results are expressed as mean ± SEM and are representative of at least two experiments. Download FIG S2, TIF file, 0.5 MB. Copyright © 2017 Costa et al. 2017 Costa et al. This content is distributed under the terms of the Creative Commons Attribution 4.0 International license.

**FIG 2:**
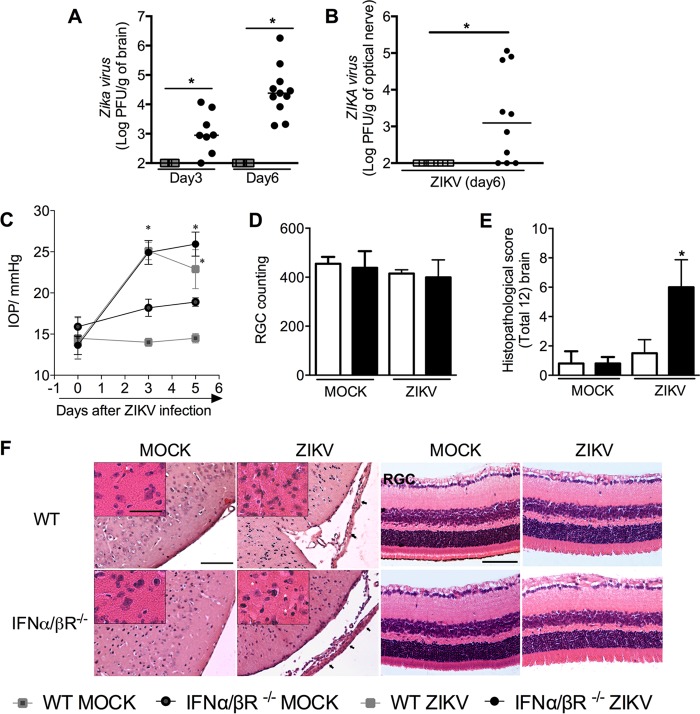
Viral load, intraocular pressure, and histopathological analysis of brain and eyes from ZIKV-infected mice. (A to F) WT (SV129) and IFN-α/βR^−/−^ mice (*n =* 8 to 11 mice per group) were inoculated i.v. with 4 × 10^5^ PFU of a Brazilian ZIKV strain (HS-2015-BA-01), and at day 3 or 6 of infection, tissues were harvested for plaque assay analysis of brain (A) and optic nerve (B). The results are shown as the log PFU per gram of brain or optic nerve, respectively. (C) Intraocular pressure (IOP) measurement after ZIKV infection. Results are expressed as millimeters of Hg increase in IOP. (D and E) Semiquantitative analysis (histopathological score) after H&E staining of retinal ganglionar cells (RGC) and brain sections of control uninfected (MOCK) and ZIKV-infected mice 6 days after infection. (F) Representative pictures from brain (left) and optic nerve (right) sections on day 6 of infection. Results are expressed as the median (A and B) or mean ± SEM (C to E) and are representative of at least two experiments. Asterisks indicate necrotic/apoptotic cells, and arrows indicate meningeal inflammation. Original magnification, ×200. Scale bar, 100 µm. Insert magnification, ×400. Inset scale bar, 50 µm.

Several studies have shown that ZIKV is neurotropic (19–22). Accordingly, studies conducted in mice detected elevated viral loads or elevated ZIKV RNA levels in the brain of adult ZIKV-infected IFN-α/βR^**−/−**^ mice, even 28 days after infection (34, 35). Our data showed that massive inflammation was found at the peak of ZIKV infection in the brain of ZIKV-infected IFN-α/βR^**−/−**^ mice, as indicated by elevated levels of MPO activity (Fig. 1E) and inflammatory mediators (Fig. 1F to I). Taking into account the virus neurotropism and its effects in the brain, we decided to search for the presence of virus in this target organ. Interestingly, elevated viral loads were recovered from the brain of ZIKV-infected IFN-α/βR^**−/−**^ mice, starting on day 3 of infection and peaking on day 6 (Fig. 2A). Additionally, ZIKV was also recovered from the optic nerve of ZIKV-infected IFN-α/βR^**−/−**^ mice (Fig. 2B). Of note, no virus was detected in the brain or optic nerve of WT mice infected with ZIKV (Fig. 2A and B).

ZIKV infection has been shown to cause substantial ophthalmic alterations in newborns of infected mothers (13) and in mice (37). To investigate possible ophthalmic alterations induced by ZIKV *in vivo*, intraocular pressure (IOP) of mice was evaluated. Results revealed significant increase in IOP on days 3 and 6 after ZIKV infection in both IFN-α/βR^**−/−**^ and WT mice in comparison to mock-infected matched controls (Fig. 2C). Nevertheless, no change in the number of retinal ganglionar cells (RGC) following ZIKV infection of either WT or IFN-α/βR^**−/−**^ mice was detected (Fig. 2D and F).

**FIG 3:**
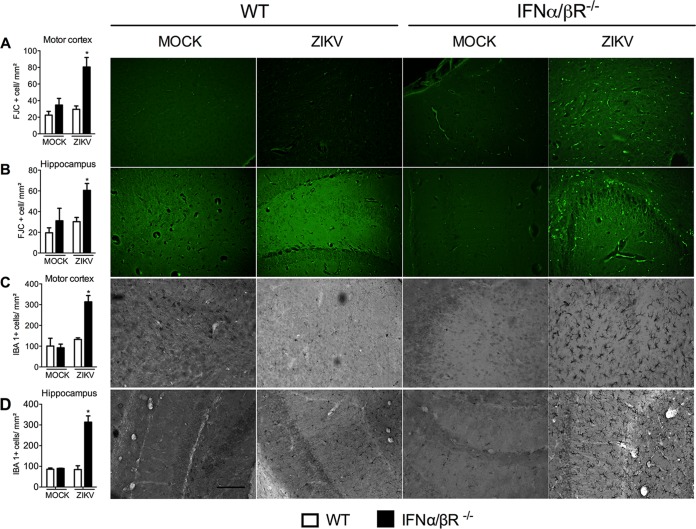
ZIKV infection induces neurodegeneration and microgliosis in the cortex and hippocampus of IFN-α/βR^−/−^ mice. (A) WT and IFN-α/βR^−/−^ mice (*n =* 5 to 7 mice per group) were inoculated i.v. with 4 × 10^5^ PFU of a Brazilian ZIKV strain (HS-2015-BA-01). At day 6 of infection, mice were culled, and brain tissue was dissected and sliced (100-µm slices). Staining with fluoro-jade C (FJC) (neurodegeneration) and IBA-I (microgliosis) was performed in cortical hippocampal slices, followed by analysis. (A and B) The left panels show the number of neurons positive for fluoro-jade C (indicative of neurodegeneration). The right panels show representative pictures from cortical and hippocampal slices. (C and D) The left panels show the number of microglia positive for IBA-1 (microgliosis). The right panels show representative pictures from cortical and hippocampal slices. All results are expressed as mean ± SEM and are representative of at least two independent experiments. *, *P* < 0.05 compared to control uninfected mice (MOCK). Original magnification, ×200. Scale bar, 50 µm.

In agreement with the inflammation (Fig. 1E to I) and elevated viral loads (Fig. 2A) found in the CNS of ZIKV-infected IFN-α/βR^**−/−**^ mice, elevated histopathological scores were observed in the brain of IFN-α/βR^**−/−**^ mice (Fig. 2E and F). Histopathological analysis of the brain of ZIKV-infected WT mice revealed mild gliosis and diffuse meningeal infiltration of polymorphonuclear and mononuclear leukocytes (Fig. 2E and F). Mock WT and IFN-α/βR^**−/−**^ mice did not show any histopathological sign of inflammation in the cerebral cortex and meninges (Fig. 2E and F). In contrast, infection of IFN-α/βR^**−/−**^ mice resulted in severe meningoencephalitis characterized by infiltration of leukocytes in the meninges (arrows), loss of hippocampal neurons, perivascular cuffs, gliosis, and focal areas with necrotic and apoptotic cells (Fig. 2E and F). Corroborating these data, severe neurodegeneration, as assessed by fluoro-jade C staining, was found in the brain of ZIKV-infected IFN-α/βR^**−/−**^ mice (Fig. 3; see Fig. S3 in the supplemental material). More specifically, ZIKV infection of IFN-α/βR^**−/−**^ mice induced significant neurodegeneration in the motor (Fig. 3A) and frontal (Fig. S3A) cortices, as well as in the hippocampus (Fig. 3B) and striatum (Fig. S3B). Microgliosis, as measured by IBA-1 staining, was also increased in different brain sections from infected IFN-α/βR^**−/−**^ mice (Fig. 3C and D; Fig. S3C and D). Therefore, the data shown here confirm the neurotropism of ZIKV, which is associated with severe brain damage in IFN-α/βR^**−/−**^ mice. Since WT mice were very resistant to ZIKV infection and showed only mild changes after virus inoculation, our next experiments were conducted only in IFN-α/βR^**−/−**^ mice, which were highly susceptible to infection.

10.1128/mBio.00350-17.3 FIG S3  ZIKV infection induces neurodegeneration and microgliosis in the prefrontal cortex and striatum of IFN-α/βR^−/−^ mice. (A) WT and IFN-α/βR^−/−^ mice (*n =* 5 to 7 mice per group) were inoculated i.v. with 4 × 10^5^ PFU of a Brazilian ZIKV strain (HS-2015-BA-01). At day 6 of infection, mice were culled, and brain tissue was dissected and sliced (100-µm slices). Staining with fluoro-jade C (FJC) (neurodegeneration) and IBA-I (microgliosis) was performed in cortical and striatal slices, followed by analysis. (A and B) The left panels show the number of neurons positive for fluoro-jade C (indicative of neurodegeneration). The right panels show representative pictures from cortical and striatal slices. (C and D) The left panels show the number of microglia positive for IBA-1 (microgliosis). The right panels show representative pictures from cortical and striatal slices. MOCK, uninfected. Original magnification, ×200. Scale bar, 50 µm. All results are expressed as mean ± SEM and are representative of at least two independent experiments. Download FIG S3, TIF file, 0.7 MB. Copyright © 2017 Costa et al. 2017 Costa et al. This content is distributed under the terms of the Creative Commons Attribution 4.0 International license.

### ZIKV induces massive neuronal damage of primary cultured neurons.

**FIG 4:**
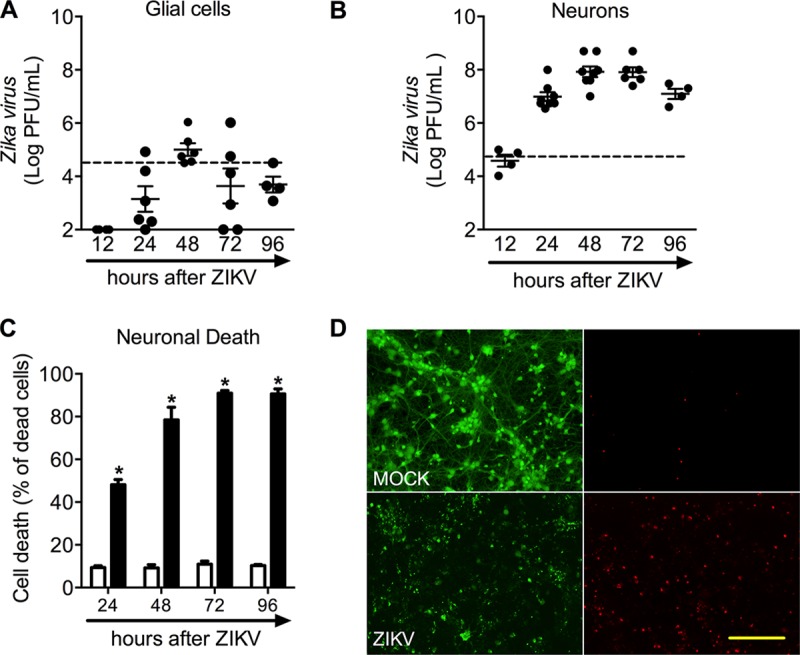
Kinetics of ZIKV infection on primary glial and neuronal cells and characterization of neuronal cell death after infection. (A) Culture of primary glial cells from a newborn C57BL/6 mouse. Viral loads were recovered from culture supernatant at different time points after infection with ZIKV (MOI of 1). (B) Primary culture of cortical-striatal neurons from C56BL/6 embryos (E15) on day 5 of differentiation *in vitro* (DIV5). The viral load was recovered from culture supernatant at different time points after infection with ZIKV (MOI of 1). (C to E) Neuronal death was assessed using the LIVE/DEAD assay in primary neurons on DIV5. (C) The panel represents the percentage of dead neurons after 12 to 96 h of infection. (D) Representative pictures from primary cultured neurons after 72 h of ZIKV infection labeled with calcein AM (green indicates live cells) and ethidium homodimer (red indicates dead cells). All results are expressed as the median (A and B) or mean ± SEM (C to E) and are representative of three to four experiments. The dashed line represents the median of remaining ZIKV titers recovered from culture supernatant at 0 h (just after the adsorption period). MOCK, uninfected. *, *P* < 0.05 compared to control uninfected neurons. Original magnification, ×200. Scale bar, 50 µm.

Mechanisms underlying cell death induced by ZIKV have not been fully elucidated. To address this question, we prepared primary cultures of glial and neuronal cells from the brain of immunocompetent mice. We evaluated the ability of ZIKV to infect and replicate in these primary cultures (Fig. 4A and B). ZIKV replication in glial cells was discrete with maximum replication levels detected at 48 h after infection, followed by reduction of viral loads at later time points (Fig. 4A). In contrast, viable virus was recovered from cell supernatant of ZIKV-infected undifferentiated neurons (5 days *in vitro*) at early time points (12 h), reaching a peak of replication at 48 and 72 h after ZIKV infection (multiplicity of infection [MOI] of 1) (Fig. 4B). Of note, virus replication in neurons was about 2- to 3-fold higher than that in glial cells, suggesting increased susceptibility of neuronal cells to ZIKV (Fig. 4A and B). Neuronal death was time dependent, reaching a peak at 72 h after ZIKV infection (Fig. 4C). Figure 4D shows representative images of mock-infected and ZIKV-infected neurons labeled with calcein acetoxymethyl ester (AM) (where green indicates live cells) and ethidium homodimer (where red indicates dead cells) after 72 h of infection. Therefore, our data show that ZIKV is able to infect and replicate in primary neurons and induce massive neuronal cell death.

### NMDAR blockade prevents neuronal death induced by ZIKV.

**FIG 5:**
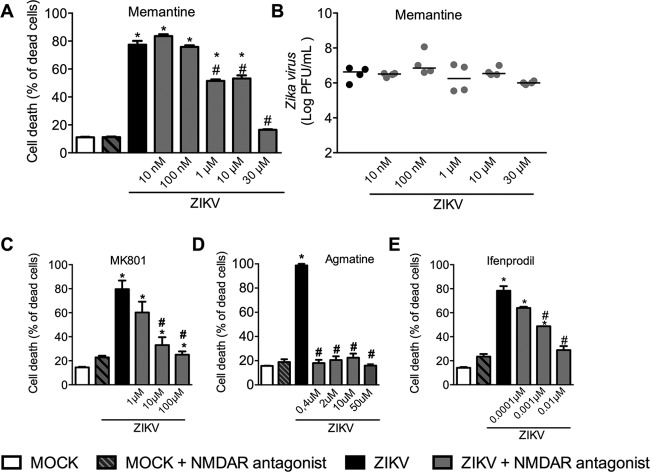
NMDAR blockade prevents ZIKV-induced neuronal cell death *in vitro*. Shown are primary cultured cortical-striatal neurons from C56BL/6 embryos (E15) on day 5 of differentiation *in vitro*. (A and B) NMDAR antagonist treatment was performed after infection (adsorption time) and every 24 h. Analyses were performed 72 h after ZIKV infection. Memantine, MK-801, and agmatine were given at different concentrations: 10 and 100 nM and 1, 10, and 30 μM for memantine, 1, 10, and 100 μM for MK-801, and 0.4, 2, 10, and 50 μM for agmatine. For ifenprodil dose-response, the concentrations were 0.0001, 0.001, and 0.01 μM. (A) Memantine’s effect on neuronal cell death was analyzed by LIVE/DEAD assay 72 h after ZIKV infection (MOI of 1). Results are represented as percentage of dead neurons. (B) Viral load was recovered from culture supernatant 72 h after ZIKV infection. Results are shown as PFU per milliliter of culture supernatant. (C) Cell death was analyzed by LIVE/DEAD assay 72 h after ZIKV infection following MK-801 treatment (MOI of 1). Results are represented as percentage of dead neurons. (D) Cell death was analyzed by LIVE/DEAD assay 72 h after ZIKV infection following agmatine sulfate treatment (MOI of 1). Results are represented as percentage of dead neurons. (E) Cell death was analyzed by LIVE/DEAD assay 72 h after ZIKV infection following ifenprodil treatment (MOI of 1). Results are represented as percentage of dead neurons. Results are expressed as mean ± SEM (A, C, and D) or the median (B) and are representative of four independent experiments. *, *P* < 0.05 compared to control uninfected neurons; #, *P* < 0.05 compared to ZIKV-infected neurons.

Neurodegeneration is a hallmark of neurodegenerative diseases such as Alzheimer’s disease (AD) (38–40). In these neurodegenerative conditions, neurological alterations appear to be closely associated with NMDAR-dependent neuronal death. Thus, we decided to test whether memantine, an NMDAR antagonist used to treat Alzheimer’s disease, can prevent neuronal cell death triggered by ZIKV infection. Our results indicate that memantine treatment was able to prevent neuronal cell death induced by ZIKV (Fig. 5A), especially at the highest tested dose (30 µM). At these concentrations, memantine did not interfere with the ability of the virus to replicate in neuronal cells *in vitro* (Fig. 5B). To further evaluate whether NMDAR blockade was the underlying mechanism of neuroprotection, we tested the effects of three other NMDAR antagonists. Dizocilpine (INN no. MK-801) is a noncompetitive antagonist of the NMDAR that, like memantine, binds inside the ion channel of the receptor, thus preventing the flow of ions, including Ca^2+^, through the channel. Figure 5C shows the dose-dependent effects of MK-801 treatment on ZIKV-infected neurons. Neuronal cell death was reduced at the 10 µM dose of MK-801 and totally prevented at the highest dose tested (100 µM). Agmatine, also known as (4-aminobutyl) guanidine, is an endogenous polyamine derived from enzymatic decarboxylation of l-arginine (41). Agmatine emerged as a neuromodulator and a promising agent to manage various central nervous system disorders by modulating the nitric oxide (NO) pathway, glutamate NMDARs, and oxidative stress (42). Figure 5D shows the effect of agmatine treatment on ZIKV-infected neurons. Interestingly, agmatine treatment prevented neuronal death in all tested doses (Fig. 5D).

NMDARs form tetrameric complexes that consist of several homologous subunits. The subunit composition of NMDARs is plastic, resulting in a large number of receptor subtypes. As each receptor subtype has distinct biophysical, pharmacological, and signaling properties, there is great interest in determining whether individual subtypes carry out specific functions in the CNS under pathological conditions (43). Transcriptome sequencing (RNA-seq) analysis performed between mock-infected and ZIKV-infected human cortical neural progenitor cells (hNPC) by Zhang et al. (44) revealed increased expression of the GRIN2B NMDAR subunit and the downstream FoxO1 gene (see Fig. S4A and B, respectively, in the supplemental material). Since primary neurons cultured for up to 9 days *in vitro* mostly express GluN2B-containing NMDARs (45), we tested whether ifenprodil, which is a specific inhibitor of GluN2B-containing NMDAR, was capable of preventing ZIKV-induced neuronal cell death. Similarly to what was observed following memantine, MK-801, and agmatine treatment, Fig. 5E shows a dose-response effect of ifenprodil in preventing ZIKV neuronal death *in vitro*. Ifenprodil treatment at a dose of 0.0001 μM has no effect on the percentage of dead cells in comparison to nontreated ZIKV-infected neurons. However, at doses of 0.001 and 0.01 μM, treatment with ifenprodil was able to partially and fully prevent ZIKV-induced neuronal death, respectively (Fig. 5E); without any change in viral load culture supernatant levels (data not shown). Together, our results show that the blockade of NMDAR prevents ZIKV-induced neuronal cell death *in vitro*.

10.1128/mBio.00350-17.4 FIG S4  ZIKV infection induces the expression of GRIN2B and FoxO1 genes in human cortical neural progenitor cells (hNPC). RNA-seq-normalization of data and differential analysis between infected and mock-infected conditions were performed by F. Zhang et al. (44). Black bars represent the expression of genes in hNPC infected with ZIKV (Asian strain FSS13025) after 64 h at an MOI of 0.04. Download FIG S4, TIF file, 0.4 MB. Copyright © 2017 Costa et al. 2017 Costa et al. This content is distributed under the terms of the Creative Commons Attribution 4.0 International license.

Memantine is a FDA-approved drug, has been widely used to treat patients with Alzheimer’s disease, and thus was the drug of choice for further investigations. To test the neuroprotective effect of memantine *in vivo*, we conducted a series of experiments in IFN-α/βR^**−/−**^ mice infected by ZIKV. Mice were treated with 30 mg/kg memantine, based on previous reports indicating that this dose was sufficient to improve a number of alterations exhibited by AD and stroke mouse models (46, 47). Memantine treatment (30 mg/kg of body weight twice a day [b.i.d.]), starting on day 3 postinfection, failed to prevent the course of certain disease manifestations induced by ZIKV infection (see Fig. S5A and S5C to G in the supplemental material). ZIKV inoculation induced body weight loss starting at day 5 of infection in both vehicle- and memantine-treated groups (Fig. S5A). In accordance, ZIKV infection induced a similar increase in MPO activity (Fig. S5C) and production of chemokines and cytokines in the brain of vehicle- and memantine-treated ZIKV-infected mice (Fig. S5D to G). However, total and differential cell counts in the blood of ZIKV-infected mice treated with memantine were significantly reduced in comparison to infected vehicle-treated littermates (Fig. S5B). Overall, memantine treatment of ZIKV-infected mice was unable to modify the course of clinical manifestations and inflammation in mouse brain.

10.1128/mBio.00350-17.5 FIG S5  Disease parameters in IFN-α/βR^−/−^ mice treated with memantine after ZIKV infection. (A to F) IFN-α/βR^−/−^ mice (*n =* 5 to 7 mice per group) were inoculated i.v. with 4 × 10^5^ PFU of a Brazilian ZIKV strain (HS-2015-BA-01). Memantine (MEM) treatment from days 2 to 6 of infection was performed orally b.i.d. (30 mg/kg per mouse). (A) Change in body weight was expressed as percentage of initial weight loss. (B to F) At the peak of infection, day 6 after ZIKV inoculation, mice were culled and blood and tissues collected for the following analysis: (B) total and differential cell counts on blood, represented as the number of differential cell counts (leukocytes, mononuclear cells, and neutrophils) normalized by percentage of total cell counts; (C) neutrophil influx into the brain, assessed by measurement of myeloperoxidase activity; (D to G) concentrations of CXCL-1, CCL5, IL-1β, and TNF-α in mouse brain, quantified by ELISA. Results are shown as picograms per 100 mg of brain tissue. All results are expressed as mean ± SEM and are representative of two independent experiments. *, *P* < 0.05 compared to control uninfected mice (MOCK); #, *P* < 0.05 compared to ZIKV-infected mice. VEIC, vehicle. Download FIG S5, TIF file, 0.8 MB. Copyright © 2017 Costa et al. 2017 Costa et al. This content is distributed under the terms of the Creative Commons Attribution 4.0 International license.

**FIG 6:**
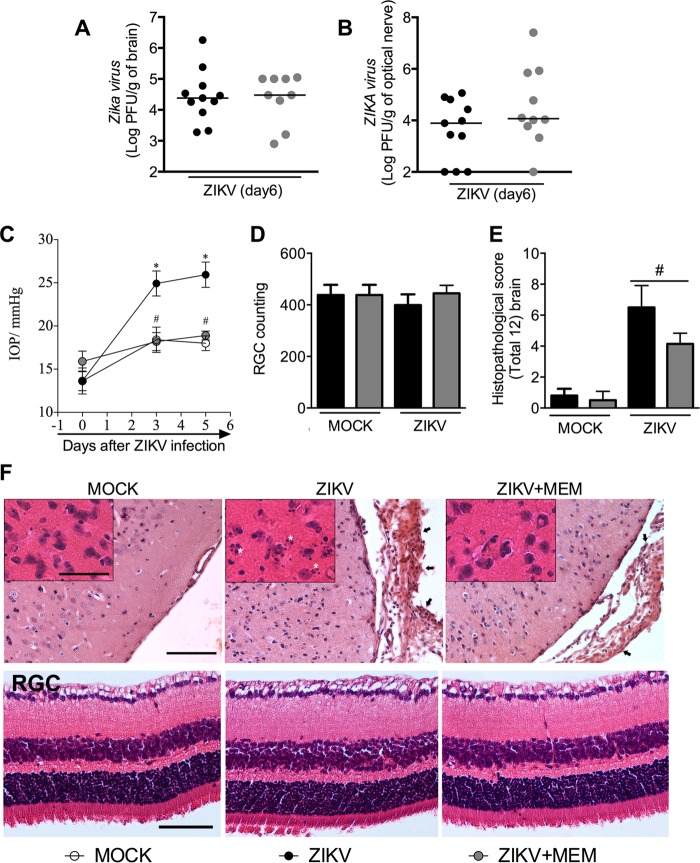
Memantine treatment prevents the increase in intraocular pressure (IOP) and brain damage induced by ZIKV. (A to F) IFN-α/βR^−/−^ mice (*n =* 5 to 11 mice per group) were inoculated i.v. with 4 × 10^5^ PFU of a Brazilian ZIKV strain (HS-2015-BA-01). Memantine (MEM) treatment (from days 3 to 6) of infection was performed orally b.i.d. (30 mg/kg). At day 6 of infection, optic nerve (B) and brain (A) were harvested for the plaque assay analysis. Results are shown as the log PFU per gram of optic nerve or brain. (C) Intraocular pressure (IOP) measurement after ZIKV infection. Results are expressed as millimeter Hg increase in IOP. (D and E) Semiquantitative analysis (histopathological score) after H&E staining of eye and brain sections of control uninfected (MOCK) and ZIKV-infected mice 6 days after infection. (F) Images are representative of each group on day 6 of infection. Results are expressed as the median (A and B) or mean ± SEM (C to E) and are representative of at least two independent experiments. *, *P* < 0.05 compared to control uninfected mice; #, *P* < 0.05 compared to ZIKV-infected mice. Asterisks indicate necrotic/apoptotic cells, and arrows indicate meningeal inflammation. Original magnification, ×200. Scale bar, 100 µm. Inset magnification, ×400. Inset scale bar, 50 µm.

**FIG 7:**
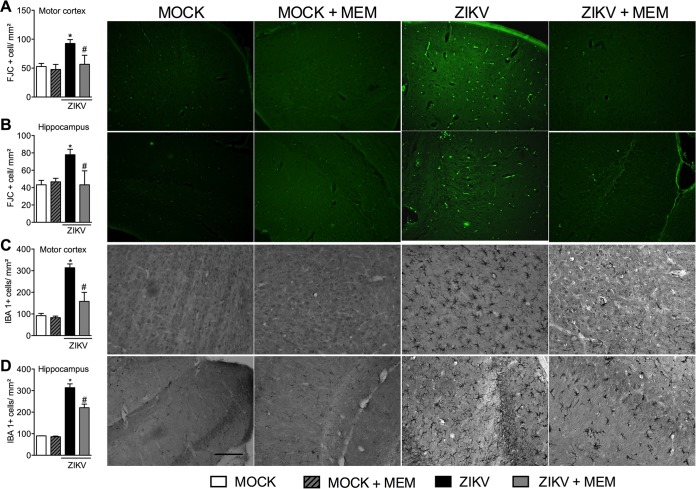
Memantine treatment prevents neurodegeneration and microgliosis induced by ZIKV infection in the cortex and hippocampus of IFN-α/βR^−/−^ mice. (A) IFN-α/βR^−/−^ mice (*n =* 5 mice per group) were inoculated i.v. with 4 × 10^5^ PFU of a Brazilian ZIKV strain (HS-2015-BA-01). Memantine (MEM) treatment from days 3 to 6 of infection was performed orally b.i.d. (30 mg/kg per mouse). At day 6 of infection, mice were culled and brain tissue was dissected and sliced (100-µm slices). (A and B) Staining with fluoro-jade C (FJC) (neurodegeneration) and (C and D) IBA-1 (microgliosis) was performed in cortical and hippocampal slices, followed by analysis. (A and B) The left panels show the number of neurons positive for fluoro-jade C (indicative of neurodegeneration). The right panels show representative pictures from cortical and hippocampal slices. (C and D) The left panels show the number of microglia positive for IBA-1 (microglial activation). The right panels show representative pictures from cortical and hippocampal slices. All results are expressed as mean ± SEM and are representative of at least two independent experiments. MOCK, uninfected. *, *P* < 0.05 compared to the uninfected group; #, *P* < 0.05 compared to the ZIKV-infected group. Original magnification, ×200. Scale bar, 50 µm.

Corroborating the *in vitro* virological data (Fig. 5B), viral loads in the brain and optic nerve of memantine-treated mice were similar to those of vehicle-treated mice (Fig. 6A and B). Interestingly, memantine treatment was also able to prevent the increase of IOP shown after ZIKV infection (Fig. 6C) without affecting the number of retinal ganglionar cells (RGC) (Fig. 6D and F). Moreover, memantine treatment partially reversed the cortical and hippocampal damage, as well as the infiltration of leukocytes in the meninges induced by ZIKV infection, as assessed by histopathological scores observed in the brain of ZIKV-infected mice compared to vehicle-treated infected controls (Fig. 6E and F). In addition to reducing overall brain damage, memantine treatment also prevented ZIKV-induced neurodegeneration (Fig. 7A and B; see Fig. S6A and B in the supplemental material) and greatly decreased microgliosis (Fig. 7C and D and Fig. S6C and D) in all brain substrates tested, including prefrontal and motor cortex, striatum, and hippocampus. Of note, no difference in all performed analyses was found between the control groups (vehicle- and memantine-treated IFN-α/βR^−/−^ mice). Overall, our results show that memantine treatment prevents neuronal cell death induced by ZIKV infection without interfering with the ability of the virus to replicate in the host.

10.1128/mBio.00350-17.6 FIG S6  Memantine prevents neurodegeneration and microgliosis induced by ZIKV infection in the prefrontal cortex and striatum of IFN-α/βR^−/−^ mice. (A) IFN-α/βR^−/−^ mice (*n =* 5 to 7 mice per group) were inoculated i.v. with 4 × 10^5^ PFU of a Brazilian ZIKV strain (HS-2015-BA-01). Memantine (MEM) treatment from days 2 to 6 of infection was performed orally b.i.d. (30 mg/kg per mouse). At day 6 of infection, mice were culled, and brain tissue was dissected and sliced (100-µm slices). (A and B) Staining with fluoro-jade C (neurodegeneration) and (C and D) IBA-I (microgliosis) was performed in cortical and striatal slices, followed by analysis. (A and B) The left panels show the number of neurons positive for fluoro-jade C (indicative of neurodegeneration). The right panels show representative images from cortical and striatal slices. (C and D) The left panels show the number of microglia positive for IBA-1 (microgliosis). The right panels show representative images from cortical and striatal slices. MOCK, uninfected. *, *P* < 0.05 compared to the uninfected group; #, *P* < 0.05 compared to the ZIKV-infected group. Original magnification, ×200. Scale bar, 50 µm. All results are expressed as mean ± SEM and are representative of at least two independent experiments. Download FIG S6, TIF file, 0.7 MB. Copyright © 2017 Costa et al. 2017 Costa et al. This content is distributed under the terms of the Creative Commons Attribution 4.0 International license.

## DISCUSSION

The rapidly expanding outbreaks of ZIKV and in particular its introduction in the Americas, including its unexpected association with neurological disorders, such as microcephaly and GBS, made this infection a matter of utmost public health concern, with an urgent need for therapies. In the present study, we evaluated the neuroprotective effect of the blockade of NMDAR by using an FDA-approved noncompetitive NMDAR antagonist drug (memantine) and other NMDAR blockers against ZIKV-associated neurodegeneration *in vitro* and *in vivo*. The major findings of this study can be summarized as follows. (i) ZIKV is able to infect and replicate in primary undifferentiated neurons and subsequently induce massive neuronal death. (ii) ZIKV induces neurodegeneration, microgliosis, and ophthalmologic disorders, such as increased intraocular pressure (IOP), *in vivo*. (iii) NMDAR blockade, by memantine, MK-801, agmatine sulfate, or ifenprodil, prevents neuronal death induced by ZIKV *in vitro* without affecting the ability of the virus to replicate in those cells. (iv) Neurodegeneration, microgliosis, and overall brain damage were ameliorated in ZIKV-infected mice, and increase in IOP levels was prevented by memantine treatment. These data, therefore, indicate an important neuroprotective effect of NMDAR blockade during ZIKV infection *in vitro* and *in vivo*.

In the present study, we characterized the disease induced by a clinical isolate of ZIKV (HS-2015-BA-01) from an outbreak that occurred in Brazil in 2015 in wild-type mice or type I or II IFN receptor-deficient mice. Our results are in accordance with the recent literature and show that IFN-α/βR^−/−^ mice are susceptible to ZIKV infection (35). As expected, WT mice exhibited no weight loss, morbidity, or mortality associated with ZIKV infection. Interestingly and unexpectedly, deficiency in the IFN-γ receptor alone did not confer increased susceptibility to infection by ZIKV, even when high inocula of the virus were administered (Fig. S1B). The finding that type II IFN alone is not sufficient to confer susceptibility in this experimental setting contrasts with our previous studies with a similar flavivirus, the dengue virus (48, 49). We also characterized the inflammatory response induced by ZIKV *in vivo*. We found that ZIKV infection induces leukocytosis, especially of lymphocytes and neutrophils, at the peak of infection. Indeed, increased leukocyte recruitment to the brain of IFN-α/βR ^−/−^ mice was detected after 6 days of infection and was associated with elevated levels of inflammatory mediators, such as the chemokines CCL5 and CXCL1 and the cytokines IL-1β and TNF-α. Interestingly, similar findings were observed in humans infected by ZIKV (50, 51). The Centers for Disease Control and Prevention (CDC) reported the occurrence of leukocytosis, with predominance of neutrophils, in a patient infected by ZIKV during an outbreak in Puerto Rico in 2015 (50), and elevated levels of inflammatory mediators such as IL-1β, IL-2, IL-4, IL-6, IL-9, IL-10, IL-13, IL-17, CCL3, CCL5, CXCL10, and vascular endothelial growth factor (VEGF) were found during both acute and convalescent phases of ZIKV (51). Those results show that ZIKV infection of IFN-α/βR^−/−^ mice mimics some features of human clinical disease and may represent a useful platform for therapeutic testing *in vivo*.

Our data support the recent evidence of ZIKV cerebral tropism and its ability to promote neurodegeneration, especially of neural progenitor cells (19–22). Here we described important histopathological alterations, such as massive neurodegeneration and microgliosis in the brain of ZIKV-infected mice. Bell and colleagues described similar results in 1971 after intracerebral infection of mice with ZIKV (52). These authors showed ZIKV replication in neurons and astroglial cells of Swiss Webster mice, and those findings were associated with cell damage, neurodegeneration, and necrosis, especially in the cortex and hippocampus (52). Accordingly, Wu and colleagues reported the occurrence of vertical ZIKV transmission experimentally, confirming the ability of ZIKV to target the radial glial cells, affecting the cortex development of offspring mice (53). Additionally, clinical evidence revealed the occurrence of ophthalmoscopic alterations in ZIKV-infected adults and infants born from ZIKV-infected mothers (13). Recently, Miner and colleagues have shown that ZIKV is able to infect several regions of the eye, including the retina, leading to apoptosis of neurons of the visual pathway (37). Our data also describes ophthalmic alterations induced by ZIKV infection *in vivo*. After infection, a significant increase in intraocular pressure (IOP) was observed in both WT and IFN-α/βR^−/−^ mice, despite the presence of ZIKV only in IFN-α/βR^−/−^ mice. However, no difference was found in RGC counts between the evaluated groups. The latter results may be explained by the short period of ZIKV infection. Our data suggest that infection by itself may be sufficient to elicit the increase of IOP levels in those mice. Corroborating our findings, Foureaux et al. demonstrated that elevated IOP levels found during the early phases of glaucoma induction in Wistar rats were independent of the reduction in the numbers of RGC, which occurred only after 15 days of glaucoma induction (54). Further studies are needed to investigate the mechanisms by which ZIKV induces the increase in IOP levels.

The development of antiviral drugs is essential for the treatment of viral infections, including by ZIKV (55). However, therapies capable of preventing the neurodegeneration induced by ZIKV may also be useful and be used in combination with antiviral drugs. Several studies indicate that activation of ionotropic glutamate receptors, especially NMDARs, plays an important role in the excitotoxic process that occurs in neurodegenerative diseases, such as Alzheimer’s disease (28). It is still not clear how ZIKV promotes neuronal cell death. However, the neurons that die due to ZIKV infection may release glutamate and promote neurodegeneration of nearby cells, propagating neuronal cell death, as typically seen in neurodegenerative processes (56). Released glutamate can also activate microglial cells and trigger neuroinflammation, which will lead to further neuronal damage (57). Additionally, brain transcriptome analysis of ZIKV-infected babies with microcephaly revealed massive alterations in glutamatergic synapse and calcium regulatory pathways (unpublished data). Our results showed that memantine treatment was able to prevent neuronal cell death and microgliosis induced by ZIKV *in vitro* and *in vivo*, without affecting the ability of ZIKV to replicate in the host. The striking effect of memantine on ZIKV-mediated neuronal cell death suggests that this NMDAR antagonist not only could be blocking propagation of neurodegeneration, as we first hypothesized, but also could be interfering with the mechanism of neuronal cell death triggered by ZIKV itself. Moreover, our results strongly indicate that ZIKV-induced neuronal cell death depends on GluN2B-conataining NMDARs. Future experiments will be important to investigate how ZIKV promotes neuronal cell death and the involvement of NMDARs in this mechanism. Since NMDAR is the major excitatory receptor in the mammalian brain, blockade of this receptor could generate a series of adverse effects due to alterations in neurotransmission. However, memantine has been shown to be safe in patients, with only minor side effects reported. For the latter reasons, memantine is the only NMDAR antagonist approved for the treatment of AD. Mechanistically, NMDAR blockade by memantine leads to a decrease in excessive calcium entry, blocking excitotoxicity (58–60). Memantine is a noncompetitive NMDAR antagonist with relatively rapid off-rate from the channel. Moreover, the effects of the drug are strongly voltage dependent, leading to selective blockade of overactivated extrasynaptic NMDARs, which are associated with neurotoxicity (61–64). Therefore, memantine blocks preferentially excessive NMDAR activity without disrupting physiological synaptic transmission, producing less adverse effects than other NMDAR antagonists (65). Notably, memantine is classified by the Food and Drug Administration (FDA) as a pregnancy drug category B, which means that memantine is probably safe to be used during pregnancy, making this drug a potential therapeutic tool to prevent and/or minimize ZIKV-related microcephaly in infected pregnant women.

In conclusion, the present study shows that ZIKV has tropism for the CNS and replicates preferentially in neurons, inducing neurodegeneration, neuroinflammation, and ophthalmologic disorders. ZIKV infection leads to massive neuronal damage via both direct replication in neuronal cells and possibly through increased excitotoxicity via overactivation of NMDARs in nearby cells. Although NMDAR blockade by memantine was very effective at preventing ZIKV-induced neuronal cell death and neurodegeneration, some aspects of disease in IFN-α/βR^−/−^ mice, as seen by body weight loss and production of inflammatory mediators, were not modified by memantine treatment. This is likely due to the very high viral loads observed in infected mice. Once effective antiviral drugs are available for *in vivo* testing, it will be important to evaluate whether the combination of neuroprotective drugs, such as memantine, and antiviral drugs could be the ideal treatment for ZIKV infection.

## MATERIALS AND METHODS

### Ethics statement.

This study was carried out in strict accordance with the regulations on ethical and animal experiments of the Brazilian Government (law 11794/2008). The experimental protocol was approved by the Committee on Animal Ethics of the Universidade Federal de Minas Gerais (CEUA/UFMG, permit protocol no. 242/2016). All surgeries were performed under ketamine/xylazine anesthesia, and all efforts were made to minimize animal suffering. Studies with ZIKV were conducted under biocontainment level 2 (BCL2) conditions at the Immunopharmacology Lab, Instituto de Ciencias Biológicas (ICB), Federal University of Minas Gerais.

### Virus.

A low-passage-number clinical isolate of ZIKV (HS-2015-BA-01), isolated from a viremic patient with symptomatic infection in Bahia State, Brazil, in 2015, was used. The complete genome of the virus is available in GenBank under accession no. KX520666. Virus stocks were propagated in C6/36 *Aedes albopictus* cells and titrated as described in reference 66. Plaques were detected after 5 days of infection.

### Mouse experiments.

For *in vivo* experiments, wild-type (WT) mice and mice deficient in type I interferon receptor (IFN-α/βR^−/−^) or type II interferon receptor (IFN-γR^−/−^), all on the SV129 background, were used. IFN-α/βR^−/−^ mice and their congenic WT controls (SV129/Ev) were originally purchased from B & K Universal Limited (United Kingdom). The IFN-γR^−/−^ strain was originally from Jackson Laboratories (reference no. 002702). All strains were obtained from Bioterio de Matrizes da Universidade de Sao Paulo—USP. Adult mice (7 to 9 weeks old) were kept under specific-pathogen-free conditions. Mice were inoculated with 4 × 10^5^ PFU ZIKV by the intravenous (i.v.) route (tail vein). Disease signs (presence of ruffled fur, partial or complete hind limb weakness or paralysis, and loss in body weight) were monitored daily. Moribund mice with 20% or more body weight loss were euthanized. This time point occurred generally between days 6 and 7 after ZIKV inoculation (the peak of ZIKV infection). In some experiments, therapeutic memantine treatment was performed orally (p.o.) at a dose of 30 mg/kg of memantine per day b.i.d. (from days 3 to 6 after ZIKV infection). For primary culture experiments (glial cells and neurons), Bioterio Central (UFMG) provided wild-type mice on the C57BL/6j (WT) genetic background.

### Hematological analysis.

Blood was obtained from the cava vein in heparin-containing syringes at the indicated times after ZIKV infection. Platelets and the hematocrit index were analyzed as described in reference 66. The total leucocyte count was obtained by using a Neubauer chamber. Differential counts were subsequently quantified microscopically from blood smears of each mouse.

### Indirect detection of leukocytes in the brain.

The extent of neutrophil accumulation in the brain of control and ZIKV-infected mice was measured by assaying myeloperoxidase activity, as previously described (67).

### Measurement of cytokine/chemokine concentrations.

The concentration of cytokines (IL-6, IL-10, TNF-α, and IFN-γ), chemokines (CXCL1 and CCL5), and growth factors (NGF and BDNF) in the brain of control and ZIKV-infected mice was measured using commercially available antibodies and according to the procedures supplied by the manufacturer (R&D Systems, Minneapolis, MN).

### Histopathological analysis.

Brains from control and ZIKV-infected mice, treated or not treated with memantine, were collected and processed for hematoxylin and eosin (H&E) staining as described in reference 67. Histopathological scoring was performed according to criteria adapted from a previous study (67) by a researcher in a blind manner. For easy interpretation, the overall score totalized 12 points. Histopathological scoring was performed in cerebral cortex and hippocampus. Each area was graded as follows: 0, no damage; 1, minimal tissue destruction and/or mild inflammation/gliosis; 2, mild tissue destruction and/or moderate inflammation/gliosis; 3, definite tissue destruction (neuronal loss and parenchymal damage) and intense inflammation; 4, necrosis (complete loss of all tissue elements with associated cellular debris). Meningeal inflammation was graded following a 0- to 4-point scale, with 0 representing no inflammation and 1 to 4 corresponding to 1 to 4 cell layers of inflammation, respectively. The final score was calculated as a sum of cerebral cortex and hippocampus scores added to the score obtained from the meningeal inflammation analysis, totalizing a maximum of 12 points.

Histopathological analysis of the eyes was performed as previously described (54). The retinal ganglion cell (RGC) counting was performed in 6 histological slides from each eye sample covering the whole extension of the retina, including the area of the optic nerve, using an Olympus BX 41 microscope (Olympus, Irving, TX).

### Immunohistochemistry IBA-1 and fluoro-jade C staining.

Sections from hippocampus, striatum, and prefrontal and motor cortex of mice were assessed for microgliosis (IBA-1 staining) according to the procedures supplied by the manufacturer (Vector Elite kit) and neurodegeneration (fluoro-Jade C staining) as described in reference 68. Results represent the analysis of 2 images that were taken from each analyzed slice. We analyzed 3 slices per brain area per mouse and used 5 to 7 mice per experiment. We first analyzed one set of mice (2 mice per group), and then we repeated the same experiment (3 to 5 animals per group) to make sure our data could be reproduced. We obtained the same results in the two independent experiments, and thus, the graphed results represent the average obtained from these 5 to 7 mice. The images presented in the article are representative of one of those experiments.

### IOP evaluation.

IOP measurements were performed on days 0, 3, and 5 after ZIKV infection using a Tono-Pen Vet applanation tonometer (Reichert Technologies, NY) as described in reference 54.

### Primary neurons and glial cell cultures.

Neuronal cultures were prepared from the cortex and striatal regions of embryonic day 15 (E15) C57BL6j wild-type mouse embryo brains, and glial cells were obtained from the whole brain of newborn C57BL6j wild-type mice (1 to 3 days). After dissection, the brain tissue was submitted to trypsin digestion followed by cell dissociation using a fire-polished Pasteur pipette. Neuronal cells were plated onto poly-l-ornithine-coated dishes in neurobasal medium supplemented with N2 and B27 supplements, 2 mM GlutaMAX, 50 μg/ml penicillin, and 50 μg/ml streptomycin, incubated at 37°C and 5% CO_2_ in a humidified incubator, and cultured for 5 days *in vitro*. Glial cells were cultured in Dulbecco’s modified Eagle’s medium (DMEM) supplemented with 10% fetal bovine serum, 1% streptomycin, and 0.3% amphotericin B and placed in 75-cm^2^ disposable culture bottles, prepared by coating with 2% gelatin, followed for 14 days of incubation at 37°C and 5% CO_2_ in a humidified incubator. Culture medium was replenishment every 4 days, and glial cells were plated onto poly-l-ornithine plates. Infection was performed with ZIKV (MOI of 1), followed by an adsorption period of 1 h. Residual virus was removed and titrated (represented by a dashed line on graphs). Wells were then washed twice with incomplete medium. Each well was replaced by a final volume of complete neurobasal medium in the presence or absence of the NMDAR antagonists. Titers are representative of virus accumulation over the whole period of infection, collected at different time points. Beside the kinetic experiments, all experiments evaluating the effects of NMDAR blockers on primary neurons were performed after 72 h of ZIKV infection. In some experiments, neuronal cultures were treated with 30 μM memantine every 24 h.

The commercially available drugs memantine (Eurofarma), MK-801 (Calbiochem), agmatine sulfate (chem-IMPEX Intl., Inc.), and ifenprodil (Tocris) were used for the *in vitro* blockade of NMDARs on primary cultured neurons.

### Cell viability by LIVE/DEAD.

Neuronal cell death was determined using a LIVE/DEAD assay kit. Briefly, neurons were stained with 2.0 µM calcein acetoxymethyl ester (AM) and 2.0 µM ethidium homodimer 1 for 15 min and the fractions of live (calcein AM-positive) and dead (ethidium homodimer 1-positive) cells were determined. Neurons were visualized by fluorescence microscopy. N1E-115 cell viability was analyzed as described previously (69).

### Titration of virus.

Virus loads in culture supernatant and mouse tissues (optic nerve and brain) were assessed by plaque assay in Vero cells as described in reference 66. Results were measured as PFU per gram of tissue weight or milliliter of supernatant.

### Statistical analysis.

Results are shown as the mean ± standard error (SEM), except for viral loads, which were expressed as the median. Body weight was converted to a percentage, and weight loss/gain was calculated by subtracting the basal levels (obtained prior to infection) from those of control and infected mice. Differences were compared using analysis of variance (ANOVA) followed by Student-Newman-Keuls *post hoc* analysis. All analyses were performed using the GraphPad PRISM software 5.0 (GraphPad Software, Inc., USA). Results with a *P* value of <0.05 were considered significant.

## References

[1] Costa VV, Fagundes CT, Souza DG, Teixeira MM 2013 Inflammatory and innate immune responses in dengue infection: protection versus disease induction. Am J Pathol 182:1950–1961. doi:10.1016/j.ajpath.2013.02.027. 23567637

[2] Musso D, Nilles EJ, Cao-Lormeau VM 2014 Rapid spread of emerging Zika virus in the Pacific area. Clin Microbiol Infect 20:O595–O596. doi:10.1111/1469-0691.12707. 24909208

[3] Ribeiro LS, Marques RE, Jesus AM, Almeida RP, Teixeira MM 2016 Zika crisis in Brazil: challenges in research and development. Curr Opin Virol 18:76–81. doi:10.1016/j.coviro.2016.04.002. 27179929

[4] Dick GW, Kitchen SF, Haddow AJ 1952 Zika virus. I. Isolations and serological specificity. Trans R Soc Trop Med Hyg 46:509–520. doi:10.1016/0035-9203(52)90042-4. 12995440

[5] Duffy MR, Chen TH, Hancock WT, Powers AM, Kool JL, Lanciotti RS, Pretrick M, Marfel M, Holzbauer S, Dubray C, Guillaumot L, Griggs A, Bel M, Lambert AJ, Laven J, Kosoy O, Panella A, Biggerstaff BJ, Fischer M, Hayes EB 2009 Zika virus outbreak on Yap Island, Federated States of Micronesia. N Engl J Med 360:2536–2543. doi:10.1056/NEJMoa0805715. 19516034

[6] Chang C, Ortiz K, Ansari A, Gershwin ME 2016 The Zika outbreak of the 21st century. J Autoimmun 68:1–13. doi:10.1016/j.jaut.2016.02.006. 26925496 PMC7127657

[7] Cao-Lormeau VM, Roche C, Teissier A, Robin E, Berry AL, Mallet HP, Sall AA, Musso D 2014 Zika virus, French Polynesia, South Pacific, 2013. Emerg Infect Dis 20:1085–1086. doi:10.3201/eid2006.140138. 24856001 PMC4036769

[8] Petersen E, Wilson ME, Touch S, McCloskey B, Mwaba P, Bates M, Dar O, Mattes F, Kidd M, Ippolito G, Azhar EI, Zumla A 2016 Rapid spread of Zika virus in the Americas—implications for public health preparedness for mass gatherings at the 2016 Brazil Olympic Games. Int J Infect Dis 44:11–15. doi:10.1016/j.ijid.2016.02.001. 26854199

[9] Zanluca C, Melo VC, Mosimann AL, Santos GI, Santos CN, Luz K 2015 First report of autochthonous transmission of Zika virus in Brazil. Mem Inst Oswaldo Cruz 110:569–572. doi:10.1590/0074-02760150192. 26061233 PMC4501423

[10] Brasil P, Pereira JP Jr, Moreira ME, Ribeiro Nogueira RM, Damasceno L, Wakimoto M, Rabello RS, Valderramos SG, Halai U, Salles TS, Zin AA, Horovitz D, Daltro P, Boechat M, Raja Gabaglia C, Carvalho de Sequeira P, Pilotto JH, Medialdea-Carrera R, Cotrim da Cunha D, Abreu de Carvalho LM, Pone M, Machado Siqueira A, Calvet GA, Rodrigues Baião AE, Neves ES, Nassar de Carvalho PR, Hasue RH, Marschik PB, Einspieler C, Janzen C, Valderramos SG, Cherry JD, Bispo de Filippis AM, Nielsen-Saines K 2016 Zika virus infection in pregnant women in Rio de Janeiro—preliminary report. N Engl J Med 375:2321–2334. doi:10.1056/NEJMoa1602412. 26943629 PMC5323261

[11] Calvet G, Aguiar RS, Melo AS, Sampaio SA, de Filippis I, Fabri A, Araujo ES, de Sequeira PC, de Mendonça MC, de Oliveira L, Tschoeke DA, Schrago CG, Thompson FL, Brasil P, Dos Santos FB, Nogueira RM, Tanuri A, de Filippis AM 2016 Detection and sequencing of Zika virus from amniotic fluid of fetuses with microcephaly in Brazil: a case study. Lancet Infect Dis 16:653–660. doi:10.1016/S1473-3099(16)00095-5. 26897108

[12] Mlakar J, Korva M, Tul N, Popović M, Poljšak-Prijatelj M, Mraz J, Kolenc M, Resman Rus K, Vesnaver Vipotnik T, Fabjan Vodušek V, Vizjak A, Pižem J, Petrovec M, Avšič Županc T 2016 Zika virus associated with microcephaly. N Engl J Med 374:951–958. doi:10.1056/NEJMoa1600651. 26862926

[13] Ventura CV, Maia M, Ventura BV, Linden VV, Araújo EB, Ramos RC, Rocha MA, Carvalho MD, Belfort R Jr, Ventura LO 2016 Ophthalmological findings in infants with microcephaly and presumable intra-uterus Zika virus infection. Arq Bras Oftalmol 79:1–3. doi:10.5935/0004-2749.20160002. 26840156

[14] Furtado JM, Espósito DL, Klein TM, Teixeira-Pinto T, da Fonseca BA 2016 Uveitis associated with Zika virus infection. N Engl J Med 375:394–396. doi:10.1056/NEJMc1603618. 27332784

[15] Brasil P, Sequeira PC, Freitas AD, Zogbi HE, Calvet GA, de Souza RV, Siqueira AM, de Mendonca MC, Nogueira RM, de Filippis AM, Solomon T 2016 Guillain-Barre syndrome associated with Zika virus infection. Lancet 387:1482. doi:10.1016/S0140-6736(16)30058-7. 27115821

[16] Cao-Lormeau VM, Blake A, Mons S, Lastère S, Roche C, Vanhomwegen J, Dub T, Baudouin L, Teissier A, Larre P, Vial AL, Decam C, Choumet V, Halstead SK, Willison HJ, Musset L, Manuguerra JC, Despres P, Fournier E, Mallet HP, Musso D, Fontanet A, Neil J, Ghawché F 2016 Guillain-Barre syndrome outbreak associated with Zika virus infection in French Polynesia: a case-control study. Lancet 387:1531–1539. doi:10.1016/S0140-6736(16)00562-6. 26948433 PMC5444521

[17] Gulland A 2016 Zika virus is a global public health emergency, declares WHO. BMJ 352:i657. doi:10.1136/bmj.i657. 26839247

[18] Ioos S, Mallet HP, Leparc Goffart I, Gauthier V, Cardoso T, Herida M 2014 Current Zika virus epidemiology and recent epidemics. Med Mal Infect 44:302–307. doi:10.1016/j.medmal.2014.04.008. 25001879

[19] Cugola FR, Fernandes IR, Russo FB, Freitas BC, Dias JL, Guimarães KP, Benazzato C, Almeida N, Pignatari GC, Romero S, Polonio CM, Cunha I, Freitas CL, Brandão WN, Rossato C, Andrade DG, Faria DP, Garcez AT, Buchpigel CA, Braconi CT, Mendes E, Sall AA, Zanotto PM, Peron JP, Muotri AR, Beltrão-Braga PC 2016 The Brazilian Zika virus strain causes birth defects in experimental models. Nature 534:267–271. doi:10.1038/nature18296. 27279226 PMC4902174

[20] Garcez PP, Loiola EC, Madeiro da Costa R, Higa LM, Trindade P, Delvecchio R, Nascimento JM, Brindeiro R, Tanuri A, Rehen SK 2016 Zika virus impairs growth in human neurospheres and brain organoids. Science 352:816–818. doi:10.1126/science.aaf6116. 27064148

[21] Li C, Xu D, Ye Q, Hong S, Jiang Y, Liu X, Zhang N, Shi L, Qin CF, Xu Z 2016 Zika virus disrupts neural progenitor development and leads to microcephaly in mice. Cell Stem Cell 19:120–126. doi:10.1016/j.stem.2016.04.017. 27179424

[22] Tang H, Hammack C, Ogden SC, Wen Z, Qian X, Li Y, Yao B, Shin J, Zhang F, Lee EM, Christian KM, Didier RA, Jin P, Song H, Ming GL 2016 Zika virus infects human cortical neural progenitors and attenuates their growth. Cell Stem Cell 18:587–590. doi:10.1016/j.stem.2016.02.016. 26952870 PMC5299540

[23] Simões E Silva AC, Moreira JM, Romanelli RM, Teixeira AL 2016 Zika virus challenges for neuropsychiatry. Neuropsychiatr Dis Treat 12:1747–1760. doi:10.2147/NDT.S113037. 27478378 PMC4951060

[24] DiFiglia M 1990 Excitotoxic injury of the neostriatum: a model for Huntington’s disease. Trends Neurosci 13:286–289. doi:10.1016/0166-2236(90)90111-M. 1695405

[25] Esposito Z, Belli L, Toniolo S, Sancesario G, Bianconi C, Martorana A 2013 Amyloid β, glutamate, excitotoxicity in Alzheimer’s disease: are we on the right track? CNS Neurosci Ther 19:549–555. doi:10.1111/cns.12095. 23593992 PMC6493397

[26] Nicoletti F, Bruno V, Copani A, Casabona G, Knöpfel T 1996 Metabotropic glutamate receptors: a new target for the therapy of neurodegenerative disorders? Trends Neurosci 19:267–271. doi:10.1016/S0166-2236(96)20019-0. 8799968

[27] Conn PJ, Pin JP 1997 Pharmacology and functions of metabotropic glutamate receptors. Annu Rev Pharmacol Toxicol 37:205–237. doi:10.1146/annurev.pharmtox.37.1.205. 9131252

[28] Mody I, MacDonald JF 1995 NMDA receptor-dependent excitotoxicity: the role of intracellular Ca^2+^ release. Trends Pharmacol Sci 16:356–359. doi:10.1016/S0165-6147(00)89070-7. 7491714

[29] Seif el Nasr M, Peruche B, Rossberg C, Mennel HD, Krieglstein J 1990 Neuroprotective effect of memantine demonstrated in vivo and in vitro. Eur J Pharmacol 185:19–24. doi:10.1016/0014-2999(90)90206-L. 2226632

[30] Anderson ER, Gendelman HE, Xiong H 2004 Memantine protects hippocampal neuronal function in murine human immunodeficiency virus type 1 encephalitis. J Neurosci 24:7194–7198. doi:10.1523/JNEUROSCI.1933-04.2004. 15306653 PMC6729180

[31] Lipton SA 1992 Memantine prevents HIV coat protein-induced neuronal injury in vitro. Neurology 42:1403–1405. doi:10.1212/WNL.42.7.1403. 1620355

[32] Müller WE, Schröder HC, Ushijima H, Dapper J, Bormann J 1992 gp120 of HIV-1 induces apoptosis in rat cortical cell cultures: prevention by memantine. Eur J Pharmacol 226:209–214. doi:10.1016/0922-4106(92)90063-2. 1426020

[33] Rytik PG, Eremin VF, Kvacheva ZB, Poleschuk NN, Popov SA, Schröder HC, Bachmann M, Weiler BE, Müller WE 1991 Susceptibility of primary human glial fibrillary acidic protein-positive brain cells to human immunodeficiency virus infection in vitro: anti-HIV activity of memantine. AIDS Res Hum Retroviruses 7:89–95. http://online.liebertpub.com/doi/abs/10.1089/aid.1991.7.89. 1707644 10.1089/aid.1991.7.89

[34] Aliota MT, Caine EA, Walker EC, Larkin KE, Camacho E, Osorio JE 2016 Characterization of lethal Zika virus infection in AG129 mice. PLoS Negl Trop Dis 10:e0004682. doi:10.1371/journal.pntd.0004682. 27093158 PMC4836712

[35] Lazear HM, Govero J, Smith AM, Platt DJ, Fernandez E, Miner JJ, Diamond MS 2016 A mouse model of Zika virus pathogenesis. Cell Host Microbe 19:720–730. doi:10.1016/j.chom.2016.03.010. 27066744 PMC4866885

[36] Zmurko J, Marques RE, Schols D, Verbeken E, Kaptein SJ, Neyts J 2016 The viral polymerase inhibitor 7-deaza-2′-C-methyladenosine is a potent inhibitor of in vitro Zika virus replication and delays disease progression in a robust mouse infection model. PLoS Negl Trop Dis 10:e0004695. doi:10.1371/journal.pntd.0004695. 27163257 PMC4862633

[37] Miner JJ, Sene A, Richner JM, Smith AM, Santeford A, Ban N, Weger-Lucarelli J, Manzella F, Rückert C, Govero J, Noguchi KK, Ebel GD, Diamond MS, Apte RS 2016 Zika virus infection in mice causes panuveitis with shedding of virus in tears. Cell Rep 16:3208–3218. doi:10.1016/j.celrep.2016.08.079. 27612415 PMC5040391

[38] Li SH, Li XJ 2004 Huntingtin-protein interactions and the pathogenesis of Huntington’s disease. Trends Genet 20:146–154. doi:10.1016/j.tig.2004.01.008. 15036808

[39] Young AB 2003 Huntingtin in health and disease. J Clin Invest 111:299–302. doi:10.1172/JCI17742. 12569151 PMC151871

[40] Crews L, Masliah E 2010 Molecular mechanisms of neurodegeneration in Alzheimer’s disease. Hum Mol Genet 19:R12–R20. doi:10.1093/hmg/ddq160. 20413653 PMC2875049

[41] Tabor CW, Tabor H 1984 Polyamines. Annu Rev Biochem 53:749–790. doi:10.1146/annurev.bi.53.070184.003533. 6206782

[42] Moretti M, Matheus FC, de Oliveira PA, Neis VB, Ben J, Walz R, Rodrigues AL, Prediger RD 2014 Role of agmatine in neurodegenerative diseases and epilepsy. Front Biosci 6:341–359. 10.2741/E710 24896210

[43] Paoletti P, Bellone C, Zhou Q 2013 NMDA receptor subunit diversity: impact on receptor properties, synaptic plasticity and disease. Nat Rev Neurosci 14:383–400. doi:10.1038/nrn3504. 23686171

[44] Zhang F, Hammack C, Ogden SC, Cheng Y, Lee EM, Wen Z, Qian X, Nguyen HN, Li Y, Yao B, Xu M, Xu T, Chen L, Wang Z, Feng H, Huang WK, Yoon KJ, Shan C, Huang L, Qin Z 2016 Molecular signatures associated with ZIKV exposure in human cortical neural progenitors. Nucleic Acids Res 44:8610–8620. doi:10.1093/nar/gkw765. 27580721 PMC5063002

[45] Hoffmann H, Gremme T, Hatt H, Gottmann K 2000 Synaptic activity-dependent developmental regulation of NMDA receptor subunit expression in cultured neocortical neurons. J Neurochem 75:1590–1599. doi:10.1046/j.1471-4159.2000.0751590.x. 10987840

[46] Minkeviciene R, Banerjee P, Tanila H 2004 Memantine improves spatial learning in a transgenic mouse model of Alzheimer’s disease. J Pharmacol Exp Ther 311:677–682. doi:10.1124/jpet.104.071027. 15192085

[47] López-Valdés HE, Clarkson AN, Ao Y, Charles AC, Carmichael ST, Sofroniew MV, Brennan KC 2014 Memantine enhances recovery from stroke. Stroke 45:2093–2100. doi:10.1161/STROKEAHA.113.004476. 24938836 PMC4142682

[48] Fagundes CT, Costa VV, Cisalpino D, Amaral FA, Souza PR, Souza RS, Ryffel B, Vieira LQ, Silva TA, Atrasheuskaya A, Ignatyev G, Sousa LP, Souza DG, Teixeira MM 2011 IFN-gamma production depends on IL-12 and IL-18 combined action and mediates host resistance to dengue virus infection in a nitric oxide-dependent manner. PLoS Negl Trop Dis 5:e1449. doi:10.1371/journal.pntd.0001449. 22206036 PMC3243710

[49] Costa VV, Fagundes CT, Valadão DF, Cisalpino D, Dias AC, Silveira KD, Kangussu LM, Ávila TV, Bonfim MR, Bonaventura D, Silva TA, Sousa LP, Rachid MA, Vieira LQ, Menezes GB, de Paula AM, Atrasheuskaya A, Ignatyev G, Teixeira MM, Souza DG 2012 A model of DENV-3 infection that recapitulates severe disease and highlights the importance of IFN-gamma in host resistance to infection. PLoS Negl Trop Dis 6:e1663. doi:10.1371/journal.pntd.0001663. 22666512 PMC3362616

[50] Thomas DL, Sharp TM, Torres J, Armstrong PA, Munoz-Jordan J, Ryff KR, Martinez-Quiñones A, Arias-Berríos J, Mayshack M, Garayalde GJ, Saavedra S, Luciano CA, Valencia-Prado M, Waterman S, Rivera-García B 2016 Local transmission of Zika virus—Puerto Rico, November 23, 2015–January 28, 2016. MMWR Morb Mortal Wkly Rep 65:154–158. doi:10.15585/mmwr.mm6506e2. 26890470

[51] Tappe D, Pérez-Girón JV, Zammarchi L, Rissland J, Ferreira DF, Jaenisch T, Gómez-Medina S, Günther S, Bartoloni A, Muñoz-Fontela C, Schmidt-Chanasit J 2016 Cytokine kinetics of Zika virus-infected patients from acute to reconvalescent phase. Med Microbiol Immunol 205:269–273. doi:10.1007/s00430-015-0445-7. 26702627 PMC4867002

[52] Bell TM, Field EJ, Narang HK 1971 Zika virus infection of the central nervous system of mice. Arch Gesamte Virusforsch 35:183–193. doi:10.1007/BF01249709. 5002906

[53] Wu KY, Zuo GL, Li XF, Ye Q, Deng YQ, Huang XY, Cao WC, Qin CF, Luo ZG 2016 Vertical transmission of Zika virus targeting the radial glial cells affects cortex development of offspring mice. Cell Res 26:645–654. doi:10.1038/cr.2016.58. 27174054 PMC4897185

[54] Foureaux G, Nogueira JC, Nogueira BS, Fulgêncio GO, Menezes GB, Fernandes SO, Cardoso VN, Fernandes RS, Oliveira GP, Franca JR, Faraco AA, Raizada MK, Ferreira AJ 2013 Antiglaucomatous effects of the activation of intrinsic angiotensin-converting enzyme 2. Invest Ophthalmol Vis Sci 54:4296–4306. doi:10.1167/iovs.12-11427. 23702784 PMC3739491

[55] Barrows NJ, Campos RK, Powell ST, Prasanth KR, Schott-Lerner G, Soto-Acosta R, Galarza-Muñoz G, McGrath EL, Urrabaz-Garza R, Gao J, Wu P, Menon R, Saade G, Fernandez-Salas I, Rossi SL, Vasilakis N, Routh A, Bradrick SS, Garcia-Blanco MA 2016 A screen of FDA-approved drugs for inhibitors of Zika virus infection. Cell Host Microbe 20:259–270. doi:10.1016/j.chom.2016.07.004. 27476412 PMC4993926

[56] Wang YC, Sanchez-Mendoza EH, Doeppner TR, Hermann DM 2017 Post-acute delivery of memantine promotes post-ischemic neurological recovery, peri-infarct tissue remodeling, and contralesional brain plasticity. J Cereb Blood Flow Metab 37:980–993. doi:10.1177/0271678X16648971. 27170698 PMC5363474

[57] Murugan M, Ling EA, Kaur C 2013 Glutamate receptors in microglia. CNS Neurol Disord Drug Targets 12:773–784. doi:10.2174/18715273113126660174. 24047523

[58] Chen HS, Lipton SA 1997 Mechanism of memantine block of NMDA-activated channels in rat retinal ganglion cells: uncompetitive antagonism. J Physiol 499:27–46. doi:10.1113/jphysiol.1997.sp021909. 9061638 PMC1159335

[59] Johnson JW, Kotermanski SE 2006 Mechanism of action of memantine. Curr Opin Pharmacol 6:61–67. doi:10.1016/j.coph.2005.09.007. 16368266

[60] Lipton SA 2006 Paradigm shift in neuroprotection by NMDA receptor blockade: memantine and beyond. Nat Rev Drug Discov 5:160–170. doi:10.1038/nrd1958. 16424917

[61] Chen HS, Pellegrini JW, Aggarwal SK, Lei SZ, Warach S, Jensen FE, Lipton SA 1992 Open-channel block of *N*-methyl-d-aspartate (NMDA) responses by memantine: therapeutic advantage against NMDA receptor-mediated neurotoxicity. J Neurosci 12:4427–4436. 1432103 10.1523/JNEUROSCI.12-11-04427.1992 PMC6576016

[62] Chen HS, Wang YF, Rayudu PV, Edgecomb P, Neill JC, Segal MM, Lipton SA, Jensen FE 1998 Neuroprotective concentrations of the *N*-methyl-d-aspartate open-channel blocker memantine are effective without cytoplasmic vacuolation following post-ischemic administration and do not block maze learning or long-term potentiation. Neuroscience 86:1121–1132. doi:10.1016/S0306-4522(98)00163-8. 9697119

[63] Léveillé F, El Gaamouch F, Gouix E, Lecocq M, Lobner D, Nicole O, Buisson A 2008 Neuronal viability is controlled by a functional relation between synaptic and extrasynaptic NMDA receptors. FASEB J 22:4258–4271. doi:10.1096/fj.08-107268. 18711223

[64] Xia P, Chen HS, Zhang D, Lipton SA 2010 Memantine preferentially blocks extrasynaptic over synaptic NMDA receptor currents in hippocampal autapses. J Neurosci 30:11246–11250. doi:10.1523/JNEUROSCI.2488-10.2010. 20720132 PMC2932667

[65] Ott BR, Blake LM, Kagan E, Resnick M, Memantine MEM-MD-11AB Study Group 2007 Open label, multicenter, 28-week extension study of the safety and tolerability of memantine in patients with mild to moderate Alzheimer’s disease. J Neurol 254:351–358. doi:10.1007/s00415-006-0374-x. 17345042

[66] Costa VV, Fagundes CT, Valadao DF, Avila TV, Cisalpino D, Rocha RF, Ribeiro LS, Ascenção FR, Kangussu LM, Celso MQ Jr, Astigarraga RG, Gouveia FL, Silva TA, Bonaventura D, Sampaio DA, Leite AC, Teixeira MM, Souza DG 2014 Subversion of early innate antiviral responses during antibody-dependent enhancement of dengue virus infection induces severe disease in immunocompetent mice. Med Microbiol Immunol 203:231–250. doi:10.1007/s00430-014-0334-5. 24723052

[67] Amaral DC, Rachid MA, Vilela MC, Campos RD, Ferreira GP, Rodrigues DH, Lacerda-Queiroz N, Miranda AS, Costa VV, Campos MA, Kroon EG, Teixeira MM, Teixeira AL 2011 Intracerebral infection with dengue-3 virus induces meningoencephalitis and behavioral changes that precede lethality in mice. J Neuroinflammation 8:23. doi:10.1186/1742-2094-8-23. 21388530 PMC3061920

[68] Schmued LC, Stowers CC, Scallet AC, Xu L 2005 Fluoro-jade C results in ultra high resolution and contrast labeling of degenerating neurons. Brain Res 1035:24–31. doi:10.1016/j.brainres.2004.11.054. 15713273

[69] Ferrari M, Fornasiero MC, Isetta AM 1990 MTT colorimetric assay for testing macrophage cytotoxic activity in vitro. J Immunol Methods 131:165–172. doi:10.1016/0022-1759(90)90187-Z. 2391427

