# Supplementary figures and images for: N-Methyl-d-Aspartate (NMDA) Receptor Blockade Prevents Neuronal Death Induced by Zika Virus Infection
Source: mBio. 2017 Apr 25;8(2):e00350-17. doi: 10.1128/mBio.00350-17 (PMC5405231; doi:10.1128/mBio.00350-17)

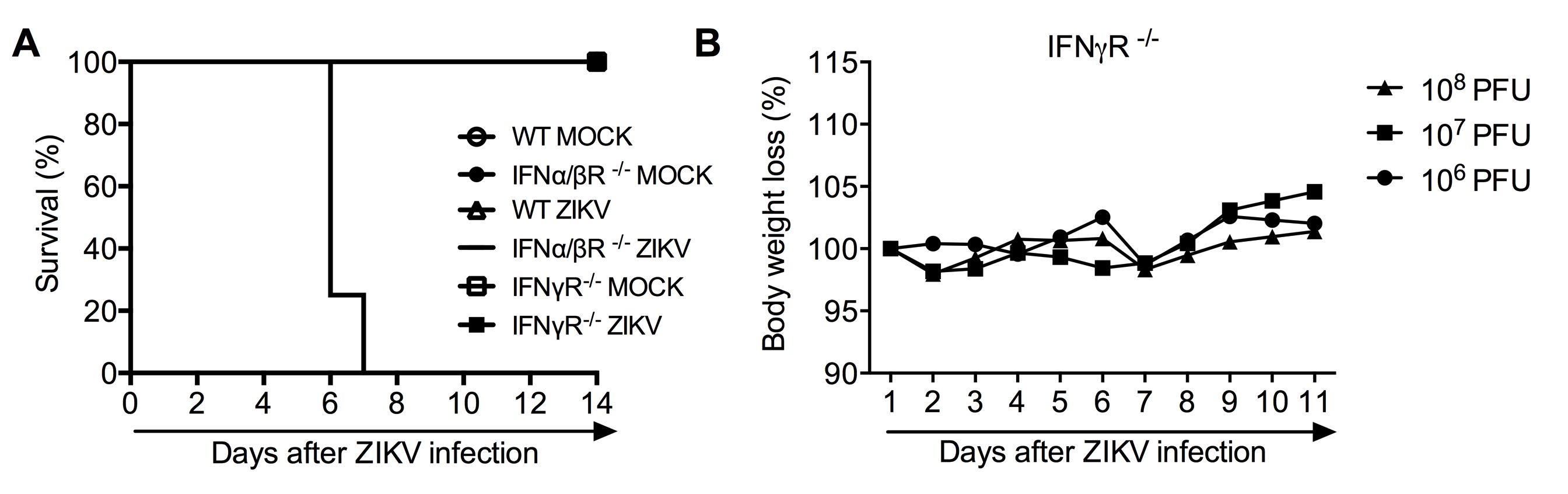

Supplement: FIG S1 [file mbo002173289sf1.tif]

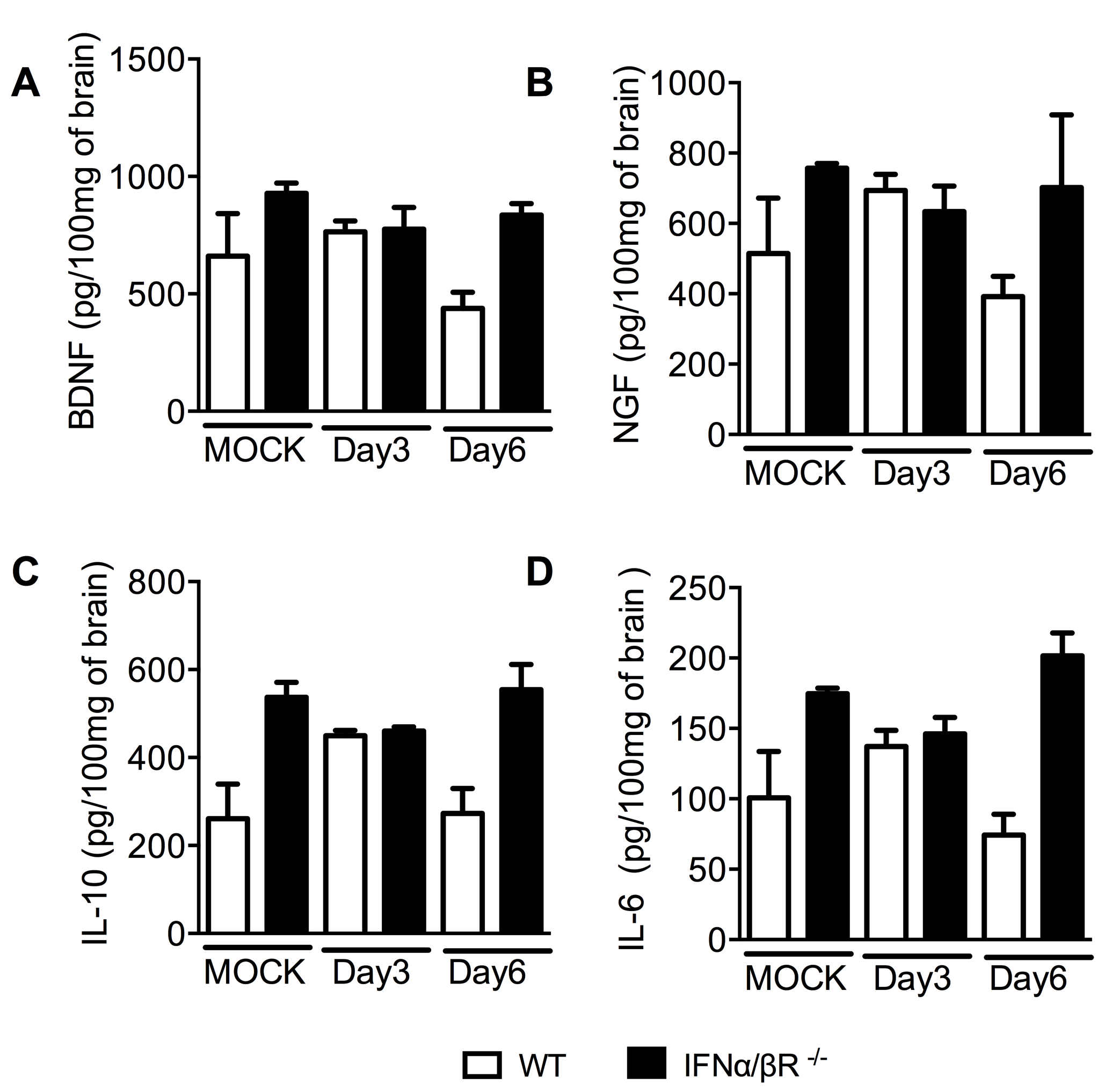

Supplement: FIG S2 [file mbo002173289sf2.tif]

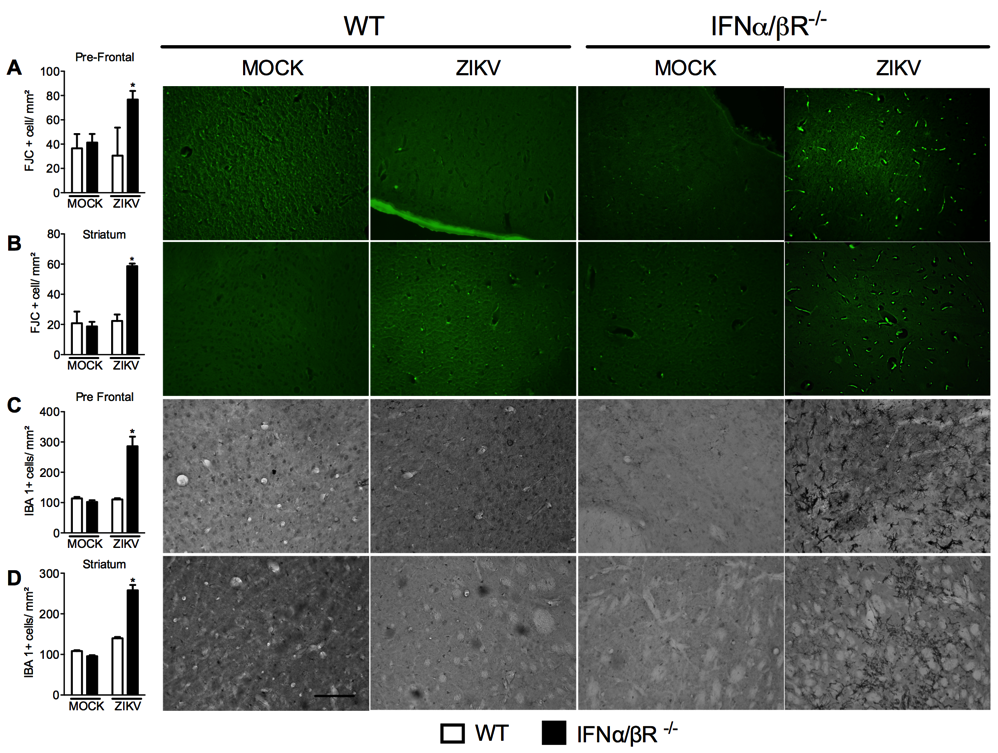

Supplement: FIG S3 [file mbo002173289sf3.tif]

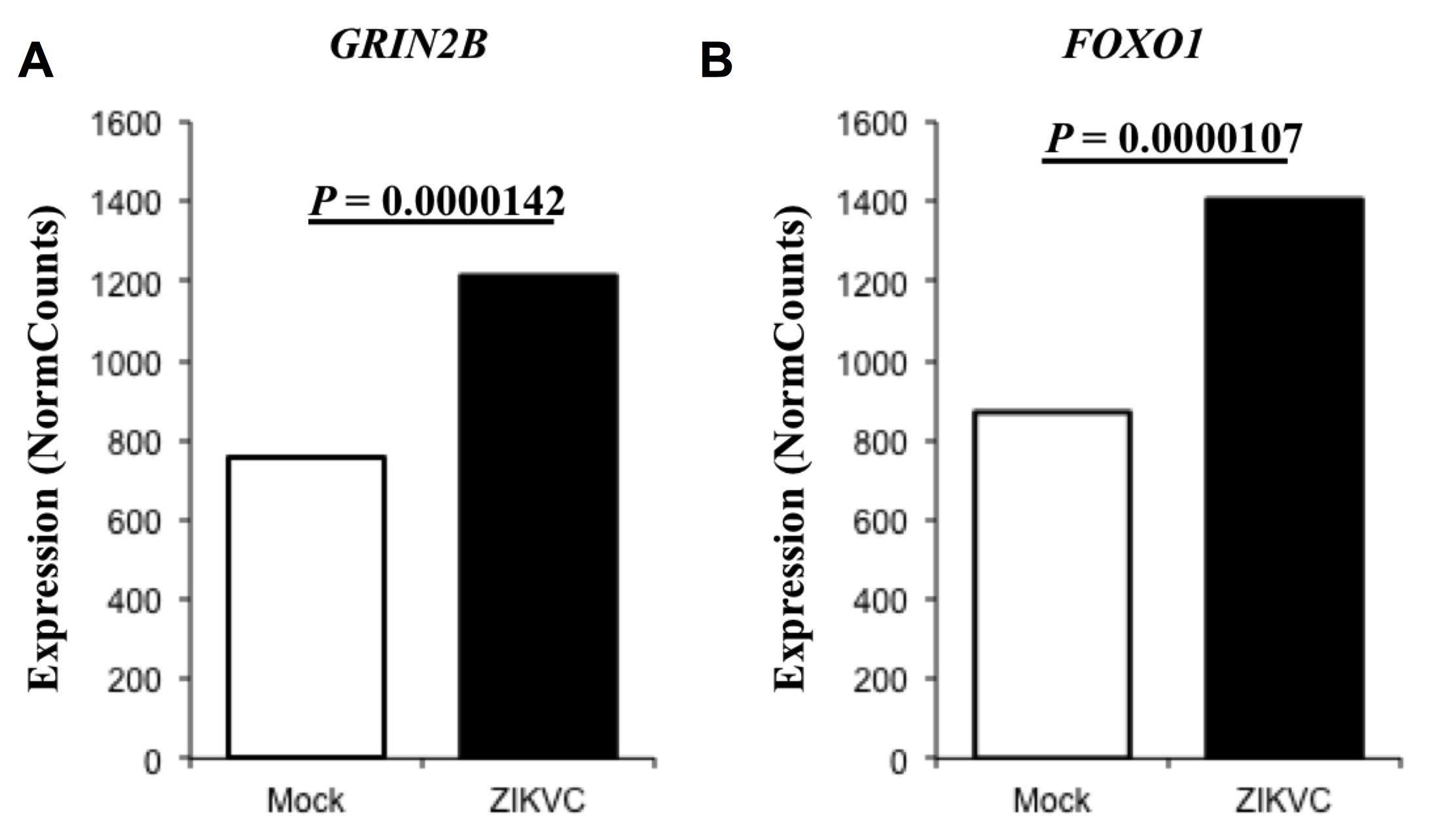

Supplement: FIG S4 [file mbo002173289sf4.tif]

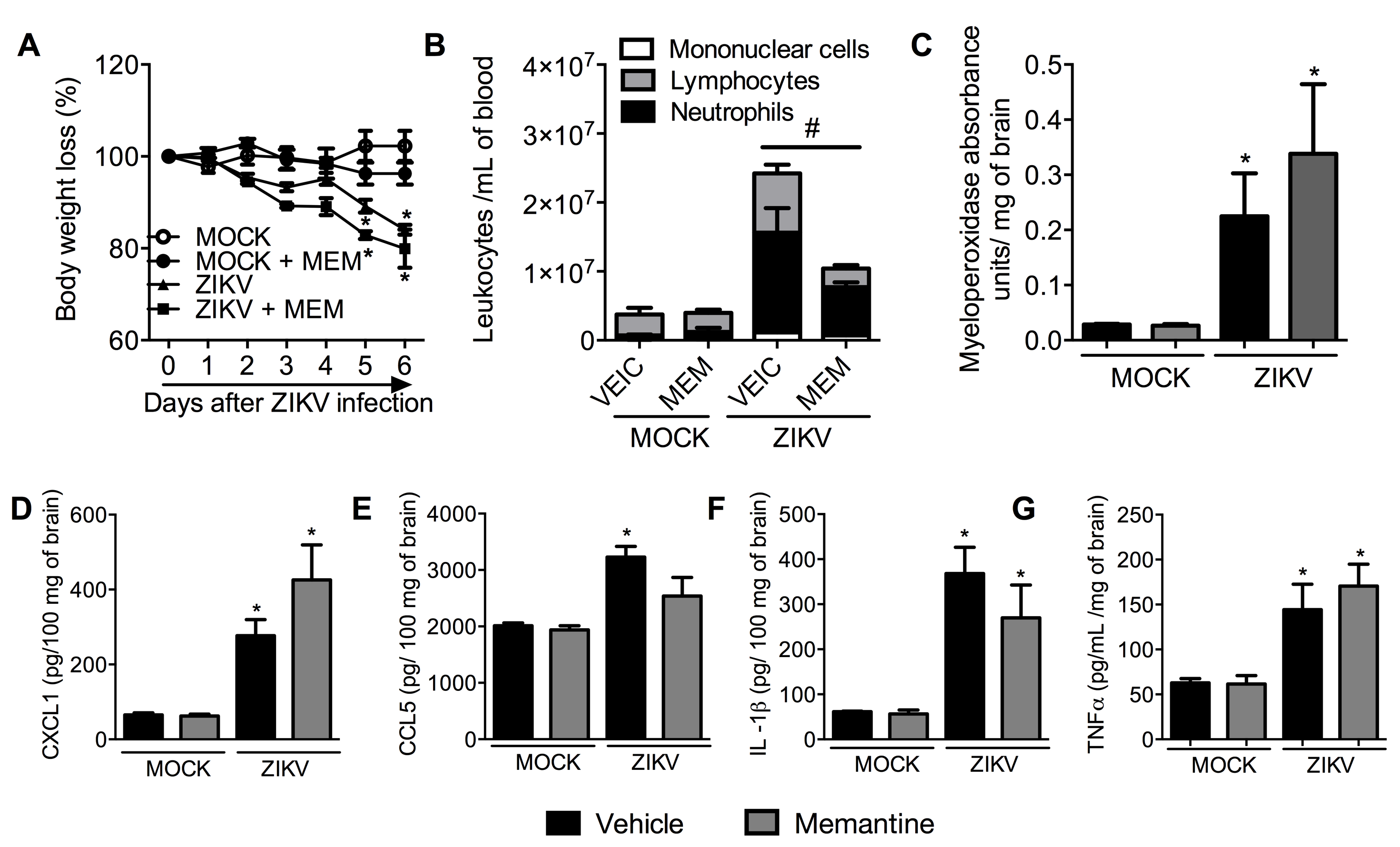

Supplement: FIG S5 [file mbo002173289sf5.tif]

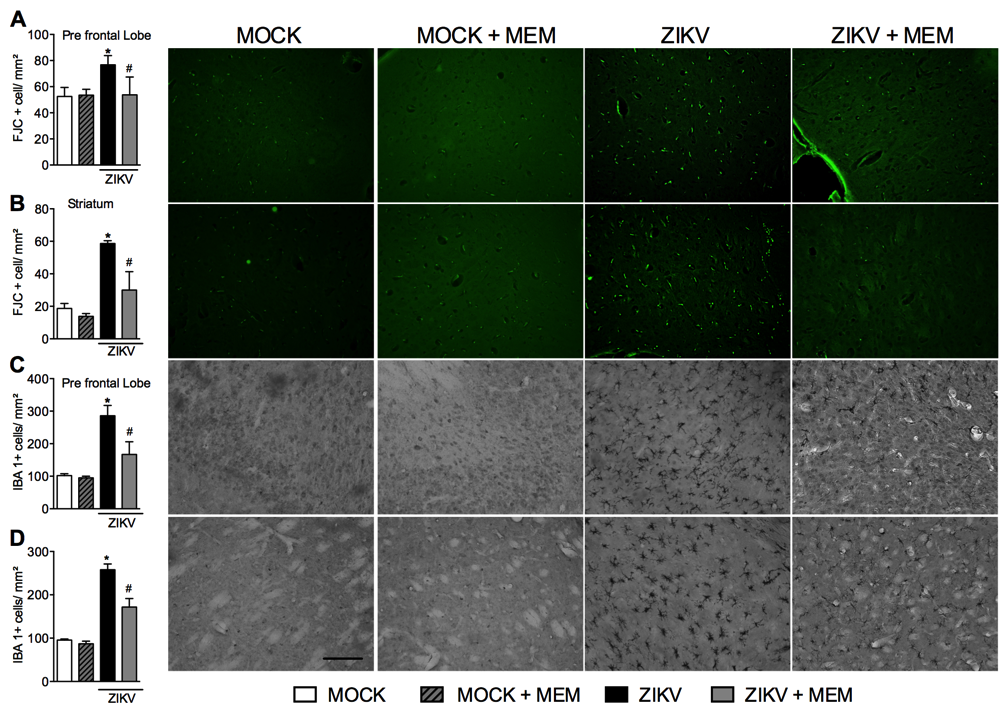

Supplement: FIG S6 [file mbo002173289sf6.tif]
